# Inferring on the Intentions of Others by Hierarchical Bayesian Learning

**DOI:** 10.1371/journal.pcbi.1003810

**Published:** 2014-09-04

**Authors:** Andreea O. Diaconescu, Christoph Mathys, Lilian A. E. Weber, Jean Daunizeau, Lars Kasper, Ekaterina I. Lomakina, Ernst Fehr, Klaas E. Stephan

**Affiliations:** 1 Translational Neuromodeling Unit (TNU), Institute for Biomedical Engineering, University of Zurich and ETH Zurich, Zurich, Switzerland; 2 Laboratory for Social and Neural Systems Research, Department of Economics, University of Zurich, Zurich, Switzerland; 3 Wellcome Trust Centre for Neuroimaging, University College London, London, United Kingdom; 4 Institut du Cerveau et de la Moelle épinière (ICM), Hôpital Pitié Salpêtrière, Paris, France; 5 Department of Computer Science, ETH Zurich, Zurich, Switzerland; University of Minnesota, United States of America

## Abstract

Inferring on others' (potentially time-varying) intentions is a fundamental problem during many social transactions. To investigate the underlying mechanisms, we applied computational modeling to behavioral data from an economic game in which 16 pairs of volunteers (randomly assigned to “player” or “adviser” roles) interacted. The player performed a probabilistic reinforcement learning task, receiving information about a binary lottery from a visual pie chart. The adviser, who received more predictive information, issued an additional recommendation. Critically, the game was structured such that the adviser's incentives to provide helpful or misleading information varied in time. Using a meta-Bayesian modeling framework, we found that the players' behavior was best explained by the deployment of hierarchical learning: they inferred upon the volatility of the advisers' intentions in order to optimize their predictions about the validity of their advice. Beyond learning, volatility estimates also affected the trial-by-trial variability of decisions: participants were more likely to rely on their estimates of advice accuracy for making choices when they believed that the adviser's intentions were presently stable. Finally, our model of the players' inference predicted the players' interpersonal reactivity index (IRI) scores, explicit ratings of the advisers' helpfulness and the advisers' self-reports on their chosen strategy. Overall, our results suggest that humans (i) employ hierarchical generative models to infer on the changing intentions of others, (ii) use volatility estimates to inform decision-making in social interactions, and (iii) integrate estimates of advice accuracy with non-social sources of information. The Bayesian framework presented here can quantify individual differences in these mechanisms from simple behavioral readouts and may prove useful in future clinical studies of maladaptive social cognition.

## Author Summary

The ability to decode another person's intentions is a critical component of social interactions. This is particularly important when we have to make decisions based on someone else's advice. Our research proposes that this complex cognitive skill (social learning) can be translated into a mathematical model, which prescribes a mechanism for mentally simulating another person's intentions. This study demonstrates that this process can be parsimoniously described as the deployment of hierarchical learning. In other words, participants learn about two quantities: the intentions of the person they interact with and the veracity of the recommendations they offer. As participants become more and more confident about their representation of the other's intentions, they make decisions more in accordance with the advice they receive. Importantly, our modeling framework captures individual differences in the social learning process: The estimated “learning fingerprint” can predict other aspects of participants' behavior, such as their perspective-taking abilities and their explicit ratings of the adviser's level of trustworthiness. The present modeling approach can be further applied in the context of psychiatry to identify maladaptive learning processes in disorders where social learning processes are particularly impaired, such as schizophrenia.

## Introduction

The process of how we represent others' intentions is an important determinant of social exchange. This inferential process becomes even more crucial when we need to rely on other people's advice regarding a course of action. Credibility can be inferred from another's reputation, which is in turn developed through recursive social interactions [1], [2]. But since advice is motivated by unknown goals, which may also change in time, we are constantly challenged by the question of how accurately we represent others' intentions.

As agents' intentions are hidden from observers, they have to be inferred from their actions. The monitoring of other agents' intentions represents a particular aspect of “theory of mind” [3]–[5]. Different cognitive frameworks for understanding this process have been suggested, e.g. action understanding vs. mentalizing (attribution of mental states) [6]–[8]. Bayesian models in particular provide a formal account of how observers build models of other agents and use them to predict their desires or intentions. One important approach is to formulate social cognition in terms of a partially observable Markov decision process (POMDP) that describes the relations between environmental states (accessible to the observer) and another agent's (unobservable) mental states [9]–[11]. This conceptualization, however, tends to be normative and does not usually emphasize individual variability in social inference. Another framework proposes that theory of mind can be understood in terms of recursive thinking, and focuses on identifying the depth of reasoning that leads to optimal inference [2], [12], [13]. Importantly, so far both types of approaches have been applied to situations where the other agents' intentions are stable over time.

In the present study we build on these previous computational treatments of how humans infer on the intentions of others by considering the additional challenge of detecting how quickly they change in time, i.e. volatility. To this end, we propose novel generative models of how humans may infer on volatile intentions of others and apply these models to behavioral data from a new experimental paradigm. The models we employ are conceptually similar to previous POMDP models, but emphasize individual approximations to Bayes-optimality, as described below.

Specifically, we addressed the following two questions by comparing the explanatory power of alternative computational models that were fitted to the observed behavior: (i) Are humans able to deploy hierarchically structured learning during social interactions and simultaneously predict the accuracy of advice and the stability of the adviser's intentions? (ii) Would humans rely more on social advice (with potentially high information but also unknown degree of uncertainty) or on non-social information that is potentially less accurate but had a known outcome distribution (i.e., risk)?

**Figure 1:**
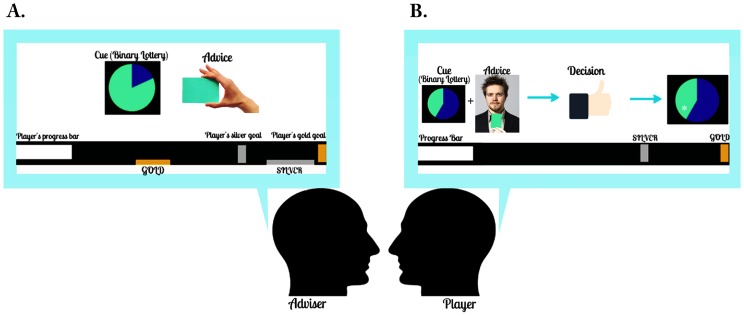
Experimental paradigm. Sixteen pairs of healthy male volunteers randomly assigned to the “adviser” role (A) or the “player” role (B) interacted in an economic game. The player had to predict the outcome of a binary lottery for which the odds were shown as a pie chart (cue). The player saw a progress bar, which increased with every correct prediction (and decreased with every incorrect/missed prediction). If the player reached the silver range, he received an extra bonus of CHF 10 (Swiss Francs); if he reached gold, he received an extra CHF 20. The adviser, however, received more information about the outcome (constant probability of 80%), and based on this information, advised the player on which option to choose. Critically, the adviser's motivation to provide valid or misleading information varied across the game. In addition to the player's progress bar, the adviser was shown his own gold and silver ranges (which the player did not see). If the player's score landed within the adviser's silver range at the end of the game, the adviser received an extra CHF 10; if the player's score landed in the adviser's golden range, the adviser earned an extra CHF 20. Importantly, before the experiment the player was informed (truthfully) that the adviser had his own undisclosed incentives and that his intentions might change during the game.

To address these questions, we designed an interactive and deception-free economic game that involved situations of both aligned and conflicting interests between participants (all male) who were randomly assigned to a “player” or an “adviser” role. In this social exchange paradigm, which builds on a previous task by Behrens et al. (2008), participants received distinct information about the probability of two possible outcomes. The player had to predict the outcome of a binary lottery whose true probability distribution was displayed as a pie chart. The adviser issued an additional recommendation (advice) to the player. The information available to the adviser was still probabilistic, but with a larger and constant probability (80%) as it was generated after the outcome had been drawn (Figure 1).

Importantly, the adviser's payment was structured such that his incentive to provide valid or misleading advice varied during the game and introduced temporal variations in aligned and conflicting interests between player and adviser. This required the player to detect changes in the adviser's intentions and adapt his own decision-making accordingly. It is not clear, however, what exact mechanism underlies adaptive behavior in this scenario: would players only track trial-wise changes in advice accuracy, or would they invoke a more complex hierarchical model, which also assumes that players track the volatility of the advisers' intentions (see [14])? Furthermore, even if the latter was the case, would volatility estimates only serve to optimize inference and learning, or would they directly impact on trial-by-trial variability of decisions?

**Figure 2:**
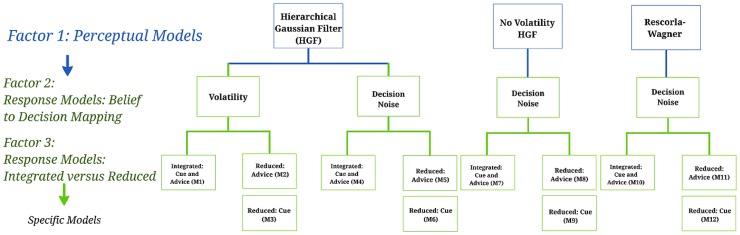
Hierarchical structure of the model space: Perceptual models, response models, specific models. The models considered in this study have a 3×2×2 factorial structure and can be displayed as a tree. The leaves at the bottom represent individual models of social learning in which both social and non-social sources of information are considered. The nodes at the first level represent the perceptual model families (three-level HGF, reduced two-level HGF, and RW). Two response models were formalized under the HGF model: decision noise in the mapping of beliefs to decisions either (1) depended dynamically on the estimated volatility of the adviser's intentions (“Volatility” model) or (2) was a fixed entity over trials (“Decision noise” model). At the third level, the response model parameters can be divided further according to the weight of social versus non-social information – these models propose that participants' beliefs are based on (1) both cue and advice information and (2) advice only. The branch on the left-hand side proposes a model in which only the given cue probabilities (i.e., the pie chart) enter the response model (Cue Probability).

To address these questions, we considered different explanations (hypotheses) for the behavior displayed by our participants, each of which was formalized as a two-component model. The first component of each model represented the player's belief updating about the causes of the advisor's behavior; we refer to this component as the “perceptual model”. The second component is the “response model”, which maps the current belief to the player's actual decision (see [15], [16]). We constructed a factorially-structured set of 12 different models (model space) by systematically combining different perceptual and response models (see Figure 2), as described in detail in the Methods section. We then fitted these models to the trial-by-trial responses of each subject using Bayesian model inversion and formally compared the plausibility of all 12 models by random effects Bayesian model selection (BMS). Altogether, this corresponds to a “meta-Bayesian” approach [15], i.e., a Bayesian treatment of Bayesian models of cognition, also known as a “doubly Bayesian” [17] or “ecumenical Bayes” [18] approach. This enabled us to identify a hierarchical generative model, which may underlie social inference in our paradigm, and whose parameter estimates predicted independent behavioral data, such as explicit ratings of the players, self-reports on strategy used by the advisers and questionnaire scores.

## Materials and Methods

### Ethics statement

All participants gave written informed consent before the study, which had received ethics approval by the local responsible authorities (Kantonale Ethikkommission, KEK 2010-0312/3).

### Participants

Thirty-two healthy male adult volunteers (age range: 19-30 years; median age = 22) participated in the study. Only men participated in this study to avoid potential gender-related confounds in the pairings of advisers and players, such as gender differences in the perception of trustworthiness (with women being perceived as generally more trustworthy than men [19]).

Participants with previous neurological or psychiatric history or who were taking medication at the time were excluded from the study. Three days before the testing session, participants received a battery of psychological questionnaires, which they had to fill out online. This included the Temperament and Character Inventory (TCI-K) [20] to measure personality traits and the Interpersonal Reactivity Index (IRI) [21] to measure empathy, perspective-taking, and theory of mind traits.

### Experimental procedure

Inspired by the paradigm of Behrens et al. (2008), we developed a deception-free and interactive economic game for monetary rewards. This paradigm involved pairs of volunteers (randomly assigned to a “player” and “adviser” role) who met each other for the first time on the day of the experiment. The player had to perform a standard probabilistic reinforcement learning task and was provided with truthful information about the *a priori* probabilities of trial-wise outcomes by a visual pie chart. The outcome was either green or blue, and all trials contained one of 6 cue types (blue:green pie charts: 75∶25, 65∶35, 55∶45, 45∶55, 35∶65, and 25∶75) (Figure 1a). The adviser, however, received more accurate information: once the outcome was determined (according to the probabilities of the visual pie chart), he was informed about the result with a constant accuracy of 80%. Based on this information, the adviser issued a recommendation to the player on which option to choose. To signal his suggestion, the adviser held up a blue or a green card (Figure 1b); these recommendations were recorded, using a video camera, for use as stimuli in future experiments. Throughout the experiment, both the player and the adviser sat across from each other and were not allowed to interact in any other way than the adviser holding up a card to indicate his suggestion.

Notably, as detailed below, the adviser's pay-off was structured such that his motivation to provide valid or misleading information varied across the game. The player therefore needed to learn about the time-varying intentions of the adviser in order to decide whether to trust him or not on any given trial. In addition to computational modeling of trial-wise choices, we obtained an explicit readout of the player's estimates by requiring him, on 8 out of the total of 200 trials, to characterize the advisers' intentions as “helpful”, “misleading”, or “uninformative”. The timing of these questions and the order of the options was randomized, but they were presented at the same times across subjects.

The player's final payment was proportional to his total score, plus a potential bonus if his score ended in a predefined silver or gold range (see Figure 1). He could track the accuracy of his predictions by monitoring a progress bar at the bottom of the screen, which increased with every correct prediction and decreased with every missed or incorrect response by 1 point. By reaching the silver or the gold target, he could win CHF 10 or CHF 20, respectively (Figure 1 and Table 1). The player was informed before the experiment that the adviser had incentives that were not necessarily aligned with his own and could vary throughout the experiment.

**Table 1:** Player and adviser incentives.

**Player**	**Silver Target**	**Gold Target**
	>65 points	>115 points
**Adviser**	**Gold Range**	**Silver Range**
	35–50	95–110

The adviser was able to monitor the player's progress, and was simultaneously shown his own opportunities to gain monetary rewards (i.e., gold and silver ranges, which were unknown to the player). Critically, the targets of the player and the adviser were arranged to create situations of shared and conflicting interests: the gold range of the adviser preceded the silver target of the player, and the silver range of the adviser also ended before the onset of the gold target of the player (see Figure 1 and Table 1).

A typical interaction between the two participants during this game unfolded in the following manner (compare Figure 1): the adviser initially had an incentive to assist the player until the latter reached the adviser's gold range. Once the players' score was within the adviser's gold range, the advisers' incentive to provide misleading advice increased. Once the player recognized this hidden change in intention and either ignored the advice or decided to bet on the opposite color, the player's progress bar was likely to exceed the adviser's gold range. Consequently, if the adviser was unable to confine the player to his (the adviser's) gold range, the next-best strategy for the adviser was to help the player with correct advice again and aim to push him into his (the adviser's) silver range. Once the player reached the adviser's silver range, the adviser had an additional incentive to mislead the player again to prevent the player from moving out of his (the adviser's) silver range.

To distinguish general inference processes under volatility from inference specific to intentionality, each pair of participants also performed a control task. To exclude temporal order effects, the sequence of the two tasks was counterbalanced across participants. In the control task, the adviser was blindfolded and issued his recommendation by picking a card from 6 separate decks placed before him by the experimenter. The blindfolding removed any intentionality by preventing that the adviser could influence what advice he was giving the player; furthermore, the adviser was unable to witness trial outcomes. The predictive accuracy of the six decks of cards was either 80% or 20%. The players were informed in advance that the card decks varied in their predictive accuracy, but not what the probabilities were nor that they were constant per each deck. However, the players could observe from which deck the card was sampled. This control condition thus closely corresponded to the main task, except for the role of intentionality: the player was required to track advice accuracy under volatility (induced by the adviser blindly switching between decks with different accuracy) and had to make trial-wise decisions how to combine the veridical information from the visual pie chart with the more informative (but volatile) advice.

Both tasks included 192 trials (plus the 8 rating trials) with an equal number of 6 cue target types (75∶25, 65∶35, 55∶45, 45∶55, 35∶65, and 25∶75 blue: green pie charts). The trial outcome was randomly drawn from these probability distributions. At the end of the study, all participants were debriefed and asked to describe the strategy that they employed during the game.

### Computational modeling

In the present study, we examined how subjects updated their beliefs about others' intentions and chose to follow or disregard their advice. For this purpose, we applied two cognitive models (which we here refer to as “perceptual models”): (i) the Hierarchical Gaussian Filter (HGF), a generic Bayesian model of learning under perceptual uncertainty and environmental volatility [22], and (ii) the Rescorla-Wagner (RW) model [23], a commonly used reinforcement learning model. In order to verify whether players really deploy hierarchical learning and infer on the volatility of the adviser's intentions, we also included a reduced (non-hierarchical) version of the HGF as control; this alternative model contained only two levels of learning (see Table 2).

**Table 2:** Prior mean and variance of the perceptual and response model parameters.

Parameter	Prior mean	Prior variance
**(i) HGF model class ** 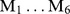		
κ	0.5	1
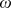	−2	100
ϑ	0.5	1
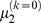	0	1
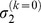	1	1
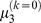	1	1
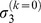	1	1
**(ii) No Volatility HGF model class** 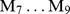		
κ	0.5	0
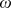	−2	100
ϑ	0.5	0
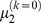	0	1
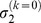	1	1
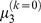	1	0
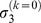	1	0
**(iii) Rescorla-Wagner model class** 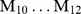		
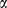	0.2	1
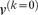	0.5	1
**(iv) Integrated model class** 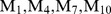		
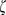	0	1
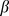	48	1
**(v) Reduced: Advice ** 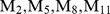		
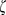	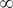	0
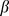	48	1
**(vi) Reduced: Cue model class ** 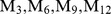		
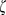	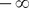	0
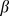	48	1

Note: The prior variances are given in the space in which parameters are estimated. 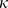, 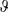 **,** 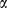,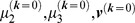 and 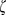 are estimated in logit-space, while 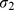 **,** 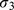 and 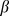 are estimated in log-space.

Furthermore, in order to link trial-by-trial beliefs to the observed decisions (and thus enable model inversion), we considered several alternative response models, which differed with regard to whether participants incorporated social and/or non-social sources of information. Together, this resulted in a factorial model space (see Figure 2), which is described in more detail below.

### Perceptual models

#### Hierarchical Gaussian Filter (HGF)

The HGF is a generic hierarchical model of learning, which allows for inference on an agent's beliefs about the state of the world from his/her observed behavior (see [22] for theoretical background and [24] and [25] for recent applications). This model is related to the “Bayesian brain” hypothesis [26]–[30], which postulates that evolutionary selection should have resulted in neural and cognitive processing principles that approximate a statistical optimum. This implies that the brain maintains and continually updates a generative (predictive) model of its sensory inputs, which allows for inference on hidden environmental states that are hierarchically organized and cause the sensory inputs that the agent experiences. In the HGF, these states evolve in time as hierarchically coupled Gaussian random walks where, at any given level, the variance (step size) is controlled by the level above (see [22] for details).

**Figure 3:**
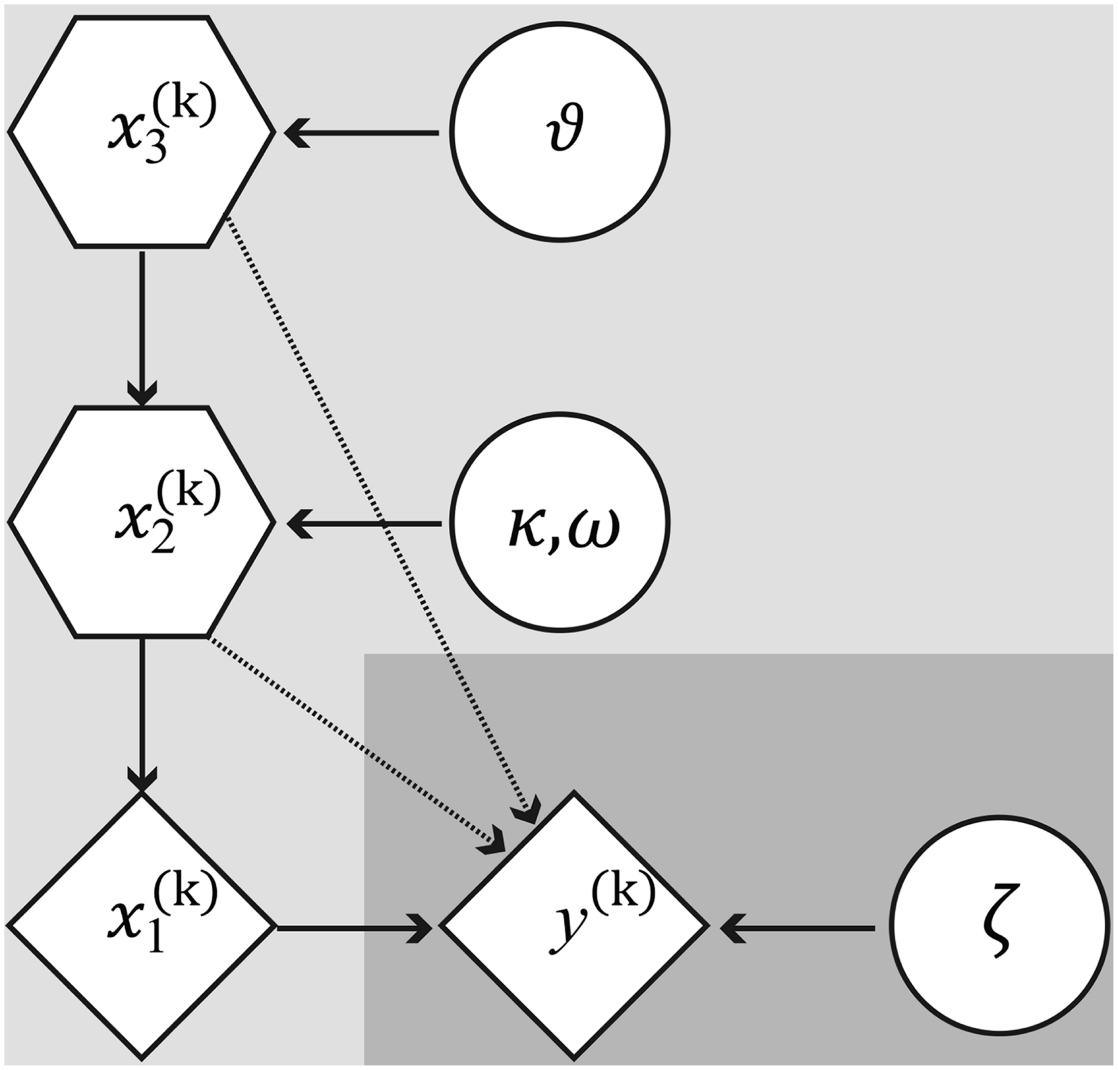
Graphical model of the HGF and the response model. In the graphical model, the diamonds represent quantities that change in time (i.e., that carry a time (or trial index 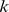) but that do not depend on their previous state. The hexagons, however, represent states that change in time but additionally depend on their previous state in time in a Markovian fashion. Circles, on the other hand, denote fixed parameters. 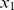 represents the accuracy of the current piece of advice, 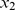 the adviser's current tendency to give accurate advice and 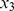 the current volatility of the adviser's intentions. Parameter 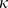 determines how strongly 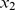 and 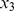 are coupled, 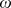 represents the tonic component of the log-volatility in 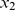 and 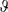 denotes the meta-volatility in 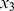. The response model has 2 layers: (1) the computation of the integrated belief or p(outcome|cued probability, advice), i.e., the probability of the outcome given both the non-social cue and the advice; (2) the chosen action, drawn from the integrated belief using a sigmoid decision rule. Parameter 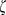 determines the weight of the advice compared to the non-social cue. 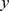 represents the subject's binary response (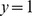: deciding to accept the advice, 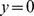: going against the advice).

In brief, the HGF proposes that an agent uses a sequence of sensory inputs to make inferences on a hierarchy of hidden states 
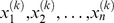
 of its environment (where *k* is a trial index and *n* is the number of levels in the hierarchy); see Figure 3. In the context of our paradigm, the player has to infer on the congruence between the advice and the outcome. Thus, the hidden state 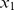 denotes the accuracy of the advice, which is binary, i.e., any single piece of advice is either accurate 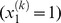 or inaccurate 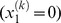.

Beliefs about advice accuracy and advisers' intentionality are represented as time-varying states in the model, where all states higher than 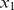 are continuous and evolve as Gaussian random walks, which are hierarchically coupled to each other in the following manner: The lowest state, 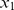 represents the participant's belief about advice accuracy, i.e., the probability that the advice is accurate (Eq. (1)), and depends on the next higher (unbounded) state 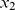 via the logistic sigmoid transformation 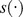 in Eq. (2).
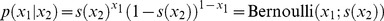
(1)where
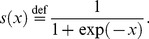
(2)


At the next higher level, 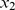 denotes the belief about the adviser's tendency to deliver accurate advice (i.e., the adviser's current degree of helpfulness). The variance or the step size with which 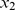 evolves over time depends on the level above, the highest state 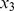.
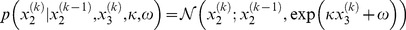
(3)


The highest state 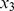 represents the (log) volatility of the adviser's intentions (i.e., tendency to offer accurate advice).
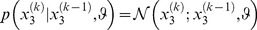
(4)


The temporal evolution of these states and their influence on each other is captured by three parameters, which can differ across agents to allow for individual belief-updating styles: (i) 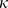 determines the degree to which the second level 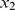 is coupled to the third level 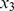, (ii) 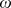 represents the constant (tonic) component of the log-volatility at the second level, which is independent of the variable (phasic) component 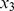, and (iii) 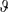 determines how quickly 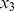 evolves over time (i.e., the step size of the Gaussian random walk performed by 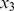). This results in the following generative model (for a graphical model of our implementation of the HGF, see Figure 3):
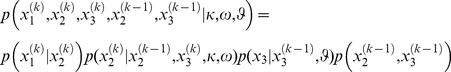
(5)


We assume that observers update their beliefs on these hierarchically-coupled states in a trial-by-trial fashion by applying an efficient approximation to ideal Bayesian inference. Under a generic mean-field approximation, such update rules have a simple and interpretable form: at each level of the hierarchy 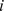, updates of beliefs (posterior means) 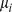 are proportional to the prediction error (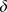) from the level below, weighted by a precision ratio:
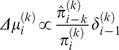
(6)where 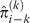 and 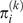 are precisions of the prediction about input from the level below and of the belief at the current level, respectively. (A note on notation: the superscript ∧ denotes the “prediction”. Hence, 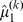 refers to the prediction on trial *k* before experiencing the trial outcome, and 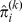 is the precision of this prediction.)

#### Rescorla-Wagner (RW) model

Reinforcement learning models propose that agents learn to take actions that maximize the probability of future rewards; therefore, agents learn the “value” of different stimuli and actions [31]. One of the most widely used reinforcement learning model is the RW model [23] where predictions about value are updated in proportion to a prediction error weighted by a learning rate. The RW model does not employ a hierarchy of hidden states, but a single state 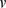 (which, in our case, describes the subject's estimated value of the advice) and one free parameter, the individual learning rate 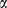, which is constant across trials:
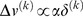
(7)


#### Structural interpretation of the HGF update equations: Analogy to the RW model

The hierarchical Bayesian model that we used to fit the data might seem complicated at first glance, but it can be reduced (via a variational approximation) to analytical update equations that have an easily interpretable form and contain only a few parameters (see [22] for details). As mentioned above, the form of these update equations is similar to those of RW learning, providing a Bayesian perspective on reinforcement learning theory [32]. Under our scheme, the general structure of these belief updates can be summarized as:
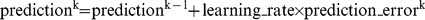
(8)


This structure is reflected by Eq. 6.

A key difference to the RW model is that the HGF uses a dynamic learning rate that is represented as a ratio of precision estimates, where at any hierarchical level 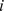, the numerator represents the (likelihood) precision of the prediction at the level below 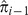, while the denominator contains the precision of the current belief, 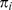. What follows from this expression is that prediction errors are given a larger weight (and thus belief updates are more pronounced) when the precision of the data (input from the lower level) is high or when the precision of the prior belief is low. This can be seen as an analogue to Kalman filtering, in the sense that the precision weighting of prediction errors corresponds to the Kalman gain.

#### Learning rates

Unlike in the RW model, the learning rate modeled in the HGF is dynamic and fluctuates trial-by-trial as a result of changes in both informational uncertainty and the volatility of the adviser's intentions. Due to the hierarchical model structure, we must consider two learning rates: the first learning rate varies as a function of perceptual and informational uncertainty and is proportional to the precision (see Eq. (9)).
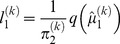
(9)where
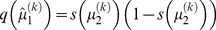
(10)


Although 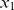 does not depend on its previous state in time but results from a sigmoid transform of 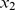 (see Eq. (2) and Figure 3), Eq. (9) transforms the learning rate at the second level into a learning rate at the first level (for more details, see Eq. D.1 in the Supplementary Material to [25]).

Furthermore, there is also a learning rate at the third level, which has a more complicated form that depends on the estimated mean 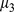 of the volatility 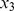 of the adviser's intentions and on the precision at the third level (for more details, see [22]):
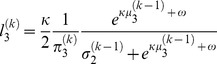
(11)where 
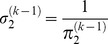
.

### Response models

The response model describes how the agent's beliefs (the result of perceptual inference) map onto choices (actions). In our task, subjects can integrate social and non-social information, or use either source of information exclusively. Specifically, the pie chart indicates the true *a priori* probability 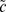 about the outcome as non-social information that is directly accessible to the player without need for inference. By contrast, the (uncertain) social information corresponds to the player's belief that the adviser gives correct advice on the current trial. In the HGF, this belief 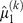 corresponds to the logistic sigmoid transform of the predicted tendency 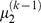 (the posterior from the previous trial) of the adviser to give correct advice (see Eq. (12)):
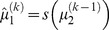
(12)


The response model describes how the player bases his decision on a weighted average of the two sources of information. Taking 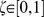 as the weight of the social information, we obtain the integrated belief that the advice is accurate:
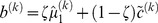
(13)


For the RW model, 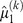 is replaced by 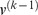.

The probability of the player following the advice (i.e., making decision 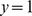 as opposed to 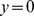 for going against the advice) is then described by a sigmoid function, which maps the unit interval 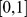 onto itself for a given decision noise parameter 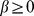 (note that this function differs from the logistic sigmoid above which maps the whole real line onto the unit interval).
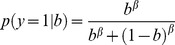
(14)


In systematic model comparisons, we compared variations in Eqs. (13) and (14), examining whether (i) subjects were more likely to integrate social and non-social information or used either source of information exclusively, and whether (ii) the decision noise in the mapping from beliefs to decisions (i.e., 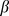 in Eq. (14)) was fixed or varied in time as a function of the estimated adviser's volatility. These variations are detailed in the section on “Model space” below.

### Model inversion

Priors of the model parameters, namely 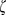 for all models as well as 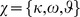 for the HGF and 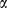 for the RW model are listed in Table 2. We defined the priors based on the experimental design and pilot data. For parameters that were strictly bounded between 0 and 1, we chose the prior mean to be 0.5. For real-valued parameters, we chose prior means that represented values under which an ideal Bayesian agent would experience the least surprise about its sensory inputs (see the functions tapas_fitModel.m and tapas_bayes_optimal_binary_config.m in the HGF toolbox). The priors were chosen to be relatively uninformative (with large variances) to allow for substantial individual differences in learning and advice weighting.

In the HGF models, we also estimated participants' initial beliefs about the advice accuracy and the adviser's volatility, as well as their uncertainty about these two quantities. Parameters and states are estimated in spaces where they are unbounded. For example, parameters confined to the [0,1] interval are log-transformed and thus also estimated in an unbounded space. Given the priors over parameters and the input sequence, maximum-a-posteriori (MAP) estimates of model parameters were calculated using the HGF toolbox version 2.1. The code used is freely available as part of the open source software package TAPAS at http://www.translationalneuromodeling.org/tapas.

Optimization was performed using a quasi-Newton optimization algorithm [33]–[36]. The objective function for maximization was the log-joint posterior density over all perceptual and observation parameters, given the data and the generative model. To exclude the possibility that our Gauss-Newton gradient descent optimization could have been influenced by local minima of the log-joint objective function, we used two additional global optimization methods, a Gaussian Process optimization algorithm (GPO) [37] and a Markov chain Monte Carlo [38] sampling scheme.

### Model space

Overall, our model space was structured hierarchically, as shown in Figure 2. We combined three alternative perceptual models with four potential response models, constituting a total of 12 models, 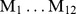, which are described in more detail below.

Although the assumptions of hierarchically coupled-learning were well founded, we also considered that participants' decisions could be explained by simpler non-hierarchical models. To examine this hypothesis, we included two model classes, which were both non-hierarchical. The first was a simplified version of the HGF (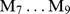), in which the volatility at the third level was fixed to its prior mean and did not evolve over time (see Table 2 for the prior values used). This model assumed that participants ignored the instructions that the advisers' intentions might change in time, expecting negligible changes in log-volatility at the third level. The second model class was the classical RW model (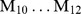), which assumed a fixed learning rate.

Concerning the response models, the key question was whether participants integrated social and non-social sources of information or relied exclusively on one of the two sources of information. Herein, we included two (reduced) response models, which proposed that participants considered either the advice alone or the cue alone when predicting the outcome. The first model was defined by setting 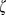 to 1 (see Eq. (15) and Table 2), whereas the second only included the displayed winning probabilities with 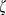 fixed to 0 (see Eq. (16) and Table 2).
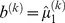
(15)

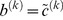
(16)


Notice that the latter response model is not coupled to any of the perceptual models, because it suggests that participants do not learn about the validity of the advice and intentions of the adviser: on the contrary, they base their decisions only on the displayed winning probabilities.

Furthermore, we assessed two potential mechanisms of belief-to-response mapping (see Eq. 14), by including models which either assumed that (i) participants responded in accordance to their belief about advice accuracy but tainted by decision noise (“Decision noise” model family for models 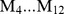), or that (ii) participants' decisions were based on their estimates of the volatility of the adviser's intentions (“Volatility” model family for models 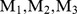).

The “Decision noise” model refers to parameter 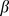 in Eq. (14), which represents the inverse of the decision temperature: as 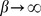, the sigmoid function becomes steeper, approaching a step function (no decision noise) at 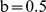. By contrast, the “Volatility” model family contains a time varying mapping of beliefs onto decisions. In this model set, the decision temperature parameter 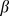 varies with the estimate of adviser volatility or 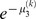. Hence, as the estimated volatility of the adviser's intentions decreases, the sigmoid function becomes steeper. This predicts that on trials when the player infers that the adviser's intentions are stable, he responds in accordance to his beliefs. As the volatility increases, the player becomes more uncertain of the adviser's intentions, and thus behaves in a more exploratory manner, resulting in a noisier mapping of belief-to-response probabilities. It is important to note that the mapping of beliefs onto actions is updated trial-wise, unlike in the case of the “Decision noise” response models, in which the link from beliefs to decisions is determined by a fixed, subject-specific parameter 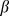. Please see the video S1 for a demonstration of how the states of the perceptual model map onto decisions using equations (13) and (14), given all possible ranges of response model parameter 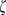.

Thanks to our factorial model space, we used family-level inference [39] to (i) determine the most likely class of perceptual models pooling across all response models, and (ii) the most likely response model class pooling across all perceptual models.

### Bayesian model selection and family inference

Before inferring on the model parameters, we evaluated the model space using Bayesian model selection (BMS). This procedure rests on computing an approximation to the model evidence or 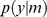 the probability of the data *y* given a model *m* [40]. The model evidence is the integral of the log-joint over the entire parameter space, which cannot be evaluated analytically. However, one can approximate the log model evidence with a lower-bound, the so-called (negative) free energy 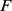.

Alternative models can then be compared via the ratio of their respective evidences, i.e. their Bayes factor or equivalently, the difference in their log-evidences. At the group level, a group Bayes factor (GBF) can be computed by multiplying Bayes factors across subjects. The disadvantage of this procedure, however, is that it rests on the fixed-effects assumption that all participants' data are generated by the same model and variation is simply due to measurement noise [41]. This is not appropriate for our paradigm, as it emphasizes individual differences in social learning (e.g., by letting advisers choose their own strategy). This requires a random-effects BMS approach, where the model becomes a random variable in the population.

The random-effects BMS approach we use here rests on a hierarchical scheme introduced by Stephan et al. (2009), which estimates the parameters 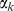 of a Dirichlet distribution of the probabilities 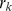 of all models considered; in turn, these probabilities inform a multinomial distribution over model space. This makes it straightforward to compute the posterior probability that a given model generated the data for any randomly selected participant, relative to all other models considered (for details see [41]). Similarly, one can compute the “exceedance probability” that a particular model is more likely than any other model in the comparison set. In other words, the exceedance probability represents the amount of evidence that, in the population studied, a given model is more frequent than the others.

In case no model really stands out as a “winner” (i.e., no high exceedance probability), we can partition the model space, pool evidence over subsets of models that share a common feature (e.g., with and without a hierarchical level) and thus compare model subspaces or families, instead of single models (see [39] for details). This idea is essentially similar to factorial experimental designs in psychology where data from all cells are used to assess the strength of main effects and interactions. It amounts to specifying a partition 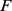, which splits the entire model set into 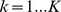 subsets (model families). The subset 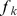 contains all models belonging to family 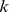 where there are 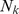 models in the 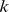 ^th^ subset. Due to the agglomerative property of the Dirichlet distribution, for any partition of model space into families, it is straightforward to define a new Dirichlet density reflecting this split (see Eq. 18 in [41]). The family probabilities are then given by:
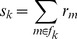
(17)where 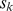 is the probability of each family occurring in the population. Exceedance probabilities 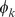 can then be computed for each family, in the same way as for single models. They correspond to the probability that family 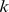 is more frequent than any other family (of all 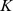 families considered), given the data 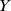 from all subjects:
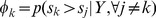
(18)


Because the conditional model probabilities 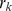 sum to one over all models considered, this equation becomes particularly intuitive when model space is split into 2 families:
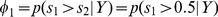
(19)


### Simulations

To test the performance of BMS for our particular case, we generated 20 datasets for each of the 4 perceptual models considered under realistic levels of decision noise and using the prior means as parameter values. Thus, we augmented the softmax function in Eq. (14), which describes the mapping of beliefs onto actions, with a noise parameter 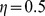:
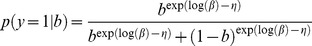
(20)


We then performed model inversion using the quasi-Newton optimization algorithm (in the same way as for the other models in this paper) and summarized the performance of BMS in terms of a confusion matrix (Figure S1). This matrix depicts the frequency of “correct cases” (where the model which generated the data has the highest exceedance probability of all models tested); here, rows denote the model that generated the data, and columns the model that was inverted. Thus, off-diagonal matrix elements indicate the frequency with which one generative model is “confused” with another.

## Results

### Examining the robustness of model inversion and comparison

As described in the Methods section, we examined the robustness of our BMS results in two ways. First, we used three different optimization schemes for inverting subject-wise models (quasi-Newton, MCMC, Gaussian process optimization). As shown by Table 3 (which lists the posterior probability of all 12 models under each optimization scheme), BMS results were consistent across all schemes. Second, the simulation results suggest that in the large majority of cases, the perceptual models that generated synthetic data could be recovered by model selection (Figure S1).

**Table 3:** BMS results across optimization schemes (posterior model probability p(r|y) of all models).

Optimization Algorithm	Response Model	HGF with Volatility	HGF with Decision Noise	No Volatility HGF	Rescorla-Wagner
1. GN	Integrated: Cue and Advice	0.78	0.0274	0.0169	0.0166
	Reduced Advice	0.0419	0.0169	0.0165	0.0171
	Reduced Cue	0.0167	0.0166	0.0168	0.0165
2. GPO	Integrated: Cue and Advice	0.6915	0.0747	0.0408	0.0190
	Reduced Advice	0.0165	0.0167	0.0279	0.0170
	Reduced Cue	0.0207	0.0178	0.0399	0.0177
3. MCMC	Integrated: Cue and Advice	0.8173	0.0165	0.0165	0.0167
	Reduced Advice	0.0164	0.0167	0.0169	0.0163
	Reduced Cue	0.0168	0.0163	0.0164	0.0172

### Do subjects exploit volatility estimates of the advisers' intentions to dynamically update estimates of advice accuracy?

**Figure 4:**
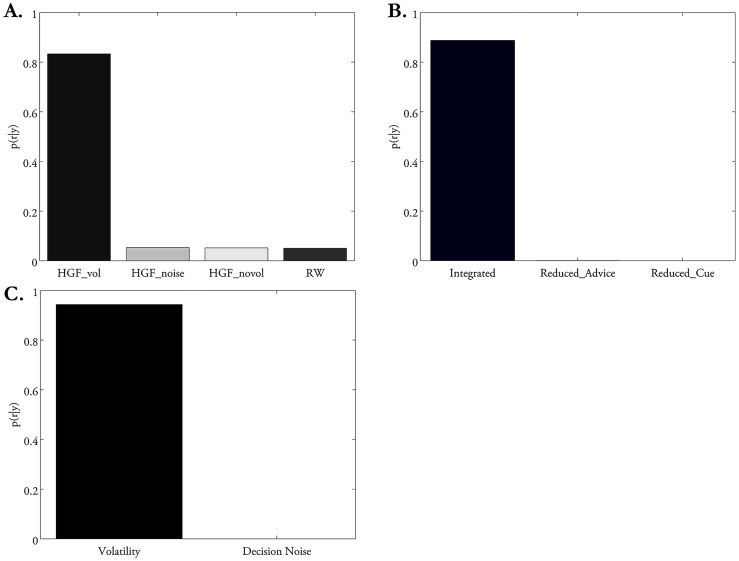
Random effects family-level Bayesian model selection. (A) Posterior model probabilities pooled across all families of perceptual model families (i.e., HGF Volatility, HGF decision noise, No Volatility HGF and RW) indicate that the model class “HGF Volatility” explains participants' responses best. (B) Posterior model probabilities pooled across all response model families (i.e., Integrated (Cue and Advice), Reduced: Advice Only, and Reduced: Cue Only) indicate that the “Integrated” model family explains participants' responses best. (C) Posterior model probabilities across models that propose that the mapping of beliefs onto response probabilities is achieved via trial-by-trial adviser volatility estimates (Volatility models) or decision noise (Decision Noise). The former was the winning family.

When comparing all 12 models against each other, random effects BMS showed that the three-level HGF augmented by the “Volatility” response model (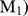 performed significantly better than the rest of the models in the majority of participants (exceedance probability 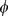 = 0.99; Table 4 and S2–S3). Across the perceptual model family, the three-level HGF family 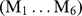 outperformed non-hierarchical models (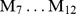) including the reduced HGF and RW models (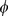 = 0.99; Table 5; Figure 4a). Taken together, these findings indicate that participants infer on two quantities, the advice accuracy and the volatility of the adviser's intentions, and incorporate the time-varying volatility estimates of the advisers' intentions into their learning about the advice.

**Table 4:** Bayesian model selection results (social interactive condition): Posterior model probability or 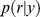 and the model exceedance probability or 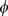.

		HGF with Volatility	HGF with Decision Noise	No Volatility HGF	Rescorla-Wagner
**Integrated**	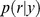	0.7800	0.0274	0.0169	0.0166
	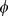	0.9975	0.0001	0	0
**Reduced: Advice**	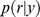	0.0419	0.0169	0.0165	0.0171
	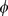	0.0005	0	0	0
**Reduced: Cue**	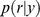	0.0167	0.0166	0.0168	0.0165
	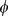	0	0	0	0

**Table 5:** Family-level inference (perceptual model set): Posterior model probability or 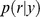 and model exceedance probabilities 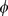.

	HGF with Volatility	HGF with Decision Noise	No Volatility HGF	Rescorla-Wagner
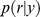	0.7298	0.1091	0.1066	0.0545
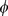	0.9969	0.0024	0.0005	0.0002

**Figure 5:**
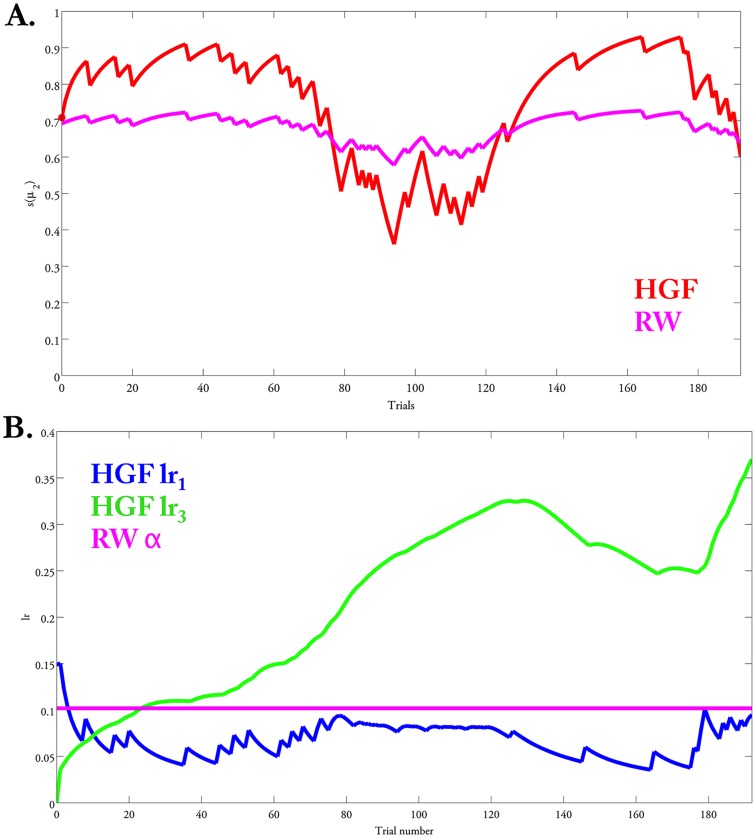
Learning rates and the estimated advice accuracy. (A) The estimated probability of the advice accuracy is computed according to the HGF and the RW perceptual models. In the RW model, the probability of advice accuracy is over- or under-estimated (compared to the HGF) on trials where the volatility of the adviser's intentions changes; this is due to the constant learning rate 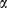. (B) The learning rates modeled in the HGF (according to equations 9 and 11) change over time as a function of the volatility of the adviser's intentions.

The HGF quantifies subject-specific learning rates at distinct temporal scales. As an example, Figure 5 contains the learning rates for one individual subject. Here, the learning rate at the second level (transformed according to Eq. 9) increases as the reliability of the advice unexpectedly changes (blue line in Figure 5). The learning rate at the third level (green line; see Eq. 11), however, fluctuates more slowly, and increases when the adviser's intentions change from being consistently helpful to being misleading. This illustrates that the learning rate adapts to fluctuations in trial-by-trial advice reliability as well as to slower fluctuations in adviser intentionality. By contrast, the RW model assumes a constant learning rate α over trials. Figure 5 contrasts this estimate of *α* with the trial-wise learning rates provided by the HGF. This comparison suggests that, in a volatile environment, such a constant learning rate is necessarily too high on many trials.

### Do subjects integrate social and non-social sources of information, or use one source of information exclusively?

The family of response models proposing that participants integrate both social and non-social sources of information (i.e., 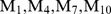) best explained participants' choices (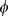 = 0.99; see Figure 4b and Table 6). That is, to predict the winning color, most participants relied on both the uncertain advice and the known outcome probabilities indicated by the visual pie chart. However, the posterior parameter estimate of 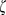 was, on average, significantly smaller than 0.5 (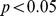; see Table 7), suggesting that participants weighted the visual pie chart information more than the advice.

**Table 6:** Family-level inference (response model set): Posterior model probability or 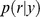 and model exceedance probabilities 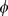.

	Integrated	Reduced: Advice	Reduced: Cue
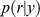	0.8740	0.0731	0.0529
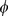	0.9998	0.0004	0.0004

**Table 7:** Average maximum a posteriori estimates of the free parameters in the winning models of the social and control tasks.

Social Interactive Task			Control Task		
Model: HGF with Volatility ( 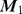 )			Model: HGF with Decision Noise ( 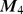 )		
Model Parameters	Mean	SD	Model Parameters	Mean	SD
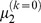	0.48	0.53	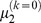	0.37	0.53
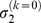	1.06	0.27	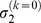	1.11	0.62
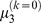	0.42	0.61	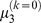	0.97	0.02
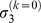	1.05	0.13	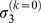	1.00	0.001
κ	0.31	0.29	κ	0.18	0.05
ω	−5.92	2.93	ω	−5.84	2.55
ϑ	0.44	0.27	ϑ	0.47	0.06
ζ	0.39	0.12	ζ	0.28	0.11
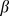	4.86	1.86	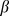	6.33	2.83

### What drives trial-by-trial variability of decisions – Estimates of volatility or general decision noise?

As explained above, we considered two possibilities vis-à-vis how the player's beliefs might determine his actions trial-by-trial. A standard approach is to use a sigmoidal function (softmax or exponentiated Luce choice rule; see Eq. 14), which conveys decision noise whose amount is fixed across trials. This approach is used in our “Decision noise” response models with a fixed, subject-specific parameter 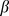. Alternatively, however, decision noise might vary dynamically across trials as a function of higher-order beliefs, such as the player's estimates of the adviser's volatility. This is represented in our “Volatility” response model family, which postulates that when the player estimates the adviser's intentions to be stable, he responds in close accordance to his beliefs. On the other hand, when the player's estimates of the adviser's volatility increase, he behaves in a more exploratory manner, resulting in a less deterministic (noisier) link between beliefs and responses. Our model comparisons indicated that the second perspective provided a better account of the data: the “Volatility” response model family clearly outperformed the “Decision noise” model family (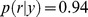; 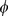 = 0.99; Figure 4c).

### Do the model parameter estimates predict other aspects of behavior?

**Figure 6:**
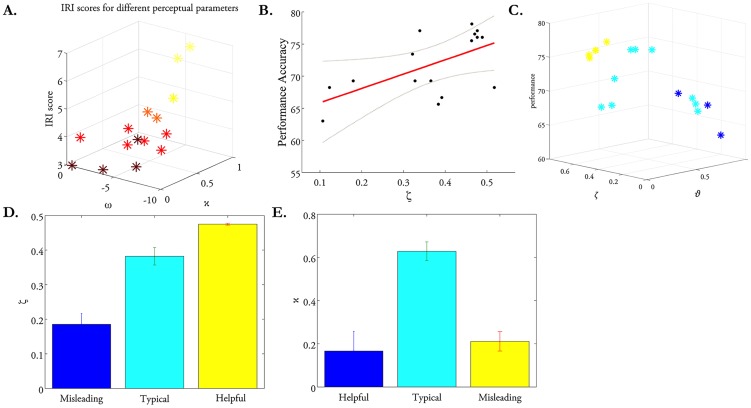
Construct validity of model parameters. The perceptual model parameter κ and ω (A) predicted players' self-report scores on the Interpersonal Reactive Index (IRI). The perceptual model parameter *ϑ* and response model parameter ζ predicted players' performance accuracy (B and C). Additionally, the perceptual model parameter 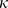 and 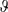 and response model parameter ζ also predicted the strategy of the advisers with whom players interacted (C–E).

We used both classical multiple regression and variational regression to examine whether model parameter estimates of the winning model (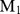) predicted scores of relevant psychological traits, as measured by questionnaires, which the subjects completed three days prior to the experimental session. Model parameter estimates 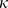 and 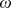 predicted players' scores on the IRI (R^2^ = 50%, F = 6.03, 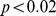; log Bayes Factor (full versus null model) = 15.16; Table 8). Thus, participants with a stronger tendency to take into account the perspective of others during social interactions showed (i) stronger coupling between inference on advice accuracy and adviser volatility and (ii) more stable belief updates about advice reliability (and adviser trustworthiness) (Figure 6a). Notably, this link between parameter estimates and independent questionnaire scores for model 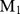 was absent for the other models (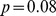 for the HGF with “Decision noise” and 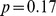 for the RW model).

**Table 8:** Predictive validity of model parameters: (a) Perceptual model parameters 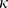 and 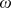 predicted participants' IRI scores.

	R^2^	F statistic	p value	Log Bayes Factor (Full versus null model)	Free Energy
**(i) Parameters ** 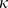 ** and ** 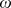 ** predict IRI scores**					
	0.50	6.03	0.02	15.16	−40.87
**(ii) Parameters** 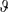 ** and ** 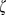 ** predict performance**					
	0.40	9.41	0.01	17.59	−146.29

Performance accuracy averaged at 73%±5% (mean ± standard deviation), indicating that, on average, the players reached the silver target and received CHF 10 bonus payment at the end of the game. Perceptual model parameter 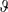 and response model parameter 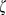 predicted participants' performance accuracy (R^2^ = 40%, F = 9.41,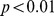; log Bayes Factor (full versus null model) = 17.59). Taken together, these results reflect that participants who perceived the adviser's intentions to be more stable and who weighted the social information more during decision-making performed better in the task (Table 8; Figure 6b–c).

### Does the player's behavior change with helpfulness of the adviser?

Advisers also reached, on average, the silver target and received CHF 10 payment at the end of the game. Across advisers, their recommendation was correct in 74%±9.8% of all trials.

On debriefing, 4 of the 16 advisers reported a general intention to help the players during the task; these advisors provided correct recommendations on 85%±9.2% of the trials (note that the information available to the advisers predicted wins with 80% accuracy). The majority of the advisers (9 out of the 16), however, aimed to increase their final pay-off and provided correct recommendations on only 74%±1.6% of the trials. On the one hand, the players who interacted with more helpful advisers weighted the advice more as indexed by larger 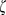 values and perceived the advisers' intentions as more stable as indexed by reduced 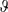 values (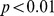; Figure 6c–d). On the other hand, the players who interacted with advisers whose intentions changed over the course of the game exhibited significantly larger 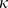 values than the rest (Figure 6e). This suggests that there was a more pronounced coupling between the two learning levels (advice accuracy and adviser volatility) during interactions with advisers whose intentions were changing.

To demonstrate the interpretability of our model parameter estimates, we asked each player eight times at random points during the game to explicitly rate the advisers as “helpful”, “uninformative” or “misleading”. These ratings were coded such that “helpful” corresponded to a probability of accurate advice of 1, “uninformative” to a probability of 0.5, and “misleading” to a probability of 0. To relate the participants' ratings to the estimates of advice reliability as inferred from the model, we used each player's ratings as the outcome variable in a general linear model with the explanatory variable being the prediction about the advice reliability (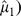. This proved to be highly significant (t = 5.92, 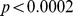) in a second level random effects regression analysis (see Figure S4). In brief, the state estimates of our model correspond well to the players' overtly expressed beliefs about the adviser's intentions during the game. Notably, the same analysis using the value of the advice as estimated by the RW model did not yield significant results (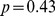). Altogether, this corroborates our model comparison results and provides construct validity for our model.

### Is behavior on the control task governed by different mechanisms?

Distinct learning performance was observed in the control task (where the adviser was blindfolded and presented his advice by holding up a card sampled from a series of card decks, each of which was, on average, either 80% or 20% accurate). The players performed significantly worse in this task compared to the socially interactive task (t (15) = 5.48, 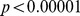), with performance accuracy averaging at 64%±2.6%.

In this task, the BMS yielded different results compared to the socially interactive task (see Table 9). More precisely, the three-level HGF family (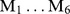) still outperformed non-hierarchical models (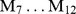), such as the reduced HGF and the RW model (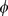 = 0.98), suggesting that participants did incorporate time-varying estimates of volatility (resulting from the switches among card decks) into their beliefs about the advice accuracy. Furthermore, the integrated response model family (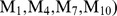, which proposed that participants weigh both social and non-social sources of information, explained participants' responses better than reduced response models (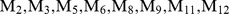) according to which subjects relied on one source of information only (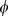 = 0.99). In contrast to the social setting, in the control task, the response model prescribing volatility-driven mapping of beliefs to decisions did not differ from the model that utilized a single decision noise parameter 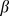
(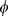 = 0.54). In other words, unlike in the social task, decision noise might not change across trials as a function of adviser volatility estimates.

**Table 9:** Results of Bayesian model selection (control condition): Posterior model probability or 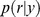 and model exceedance probabilities 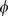.

		HGF with Volatility	HGF with Decision Noise	No Volatility HGF	Rescorla-Wagner
**Integrated**	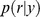	0.3187	0.3516	0.0184	0.0698
	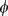	0.4112	0.5422	0.0001	0.0049
**Reduced: Advice**	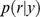	0.0166	0.0168	0.0178	0.0165
	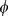	0.0001	0.0005	0.0003	0.0003
**Reduced: Cue**	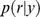	0.0179	0.0696	0.0170	0.0692
	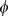	0	0.0198	0.0002	0.0204

With respect to the posterior parameter estimates (see Table 7), there were notable differences between the two tasks: In the control task, parameter 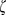 averaged at 0.28±0.11; this was significantly lower than in the social task (t (15) = 2.44, 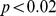), indicating that the players weighted the social (but unintentional) advice significantly less than in the social task. That is, although the card decks were more informative (80% predictability of wins/losses) than the non-social cue (55–75% predictability), the players relied more on the binary lottery information to predict the outcome. The difference in the performance and the response model parameters of these two tasks suggests that participants performed better and relied more on the adviser's recommendations when the adviser *intentionally* issued the advice.

### Differences in advisers' strategies and players' individual learning trajectories

**Figure 7:**
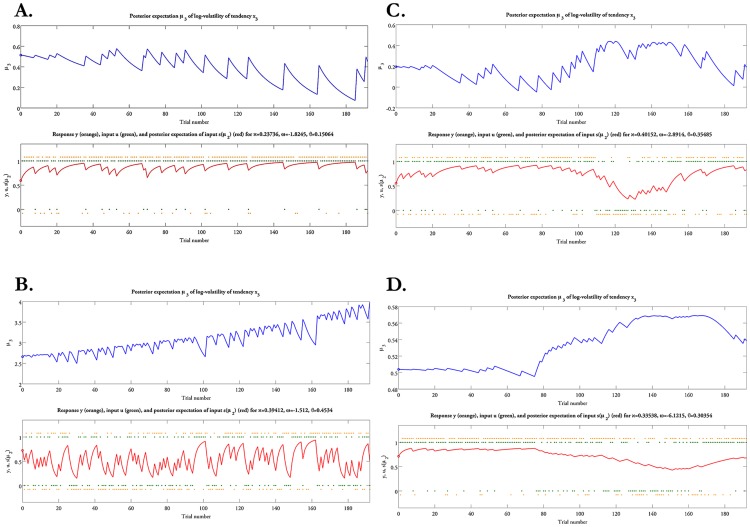
The learning trajectories about advice accuracy and adviser volatility in several representative participants. (A) Subject SL_010 interacted with a consistently helpful adviser; the parameter estimate of ζ was 0.54, suggesting that he took into account the advice more than the non-social cue. (B) Subject SL_005 interacted with a consistently misleading adviser; the estimate of ζ was 0.12, indicating that he relied almost exclusively on the non-social cue when predicting the outcome. Additionally, high levels of 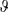 indicate that the player perceived the advice as highly volatile over the course of interaction. (C) Subject SL_013 interacted with an adviser who provided helpful advice at the beginning of the game, and then changed his strategy half-way through the game offering misleading advice. This player adapts to changes in his environment, i.e., to the advice accuracy and the adviser volatility. (D) Subject SL_015 interacted with an adviser who employed a similar strategy as in (C); however, the estimate of ω was significantly reduced suggesting that the player did not greatly change his beliefs during the interaction because his prior estimates were consistent with the adviser's actual strategy.

In each participant, the model parameters describe an individual learning trajectory (see Figure 7). As we debriefed each adviser explicitly about the strategy that he employed during the task, we were able to use these debriefings to examine how model-based quantification of individual learning and inference reflected the players' adaptive responses to the advisers' behavior during the game.

Four out of the 16 advisers provided accurate advice throughout the game with advice reliability averaging at 85%±9.2%. Upon debriefing, they reported that they aimed for the silver range from the beginning, as they deemed it fair for both participants to reach the silver target. The players who interacted with this subset of advisers perceived their intentions to be stable over time and weighted the advice more, as indexed by larger 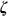 values (see Figure 6d). An example of such a player is given in Figure 7a (subject SL_010), where the trajectory of estimated advice reliability 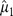 indicates that this player's estimate of advice accuracy stayed close to 90% throughout the game. For this subject, the estimate of 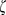 was 0.54, indicating that he relied more on the advice than the non-social cue when making his predictions.

By contrast, another 3 of the 16 advisers were consistently uninformative, exhibiting an average of only 56%±6.6% advice reliability. Indeed, when they were debriefed, they described that in order to reach the gold range, they attempted to confuse the player from the beginning, preventing his progress bar to increase significantly throughout the game and maximising their own chances to reach gold. This turned out to be a successful strategy, as the advisers who used this strategy were the only ones who reached the gold target. The players who interacted with this subset of advisers showed a very distinct trajectory of learning from those discussed above. First, 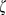 was low in all these players averaging at 0.19, indicating that they (rightfully) assigned little weight to the advice and relied exclusively on the pie chart. A representative subject is shown in Figure 7b (subject SL_005), for whom the estimate of 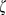 was 0.12. Furthermore, a high value of meta-volatility 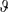 indicated that this participant perceived the adviser's intentions as becoming more and more volatile over the course of the interaction. Additionally, high levels of 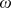 indicated fast updating of beliefs about advice correctness over trials and independently of estimates of adviser volatility. Again, this is a sign of adaptive behavior: because the adviser is consistently uninformative (random) in his advice, a high tonic learning rate for updating estimates about advice validity in 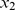 is appropriate. In other words, this scenario describes an agent who perceives the adviser's intentions as stochastic because the advice is uninformative. In this scenario, the participant necessarily performs poorly because he must base his decisions almost exclusively on the non-social cue.

The largest subset of advisers (9 out of 16) used a strategy, which reflected the change in incentives induced by the payoff scheme: advisers were helpful at the beginning of the game until the players' progress bar reached their gold range. From this point on, advisers began to mislead the players, preventing them from moving beyond the gold range. Once the players detected this change in intentionality, their score began increasing again. This elicited another switch in the advisers' strategy, who now resumed a helpful attitude in order to at least reach the silver range. On average, the recommendations of these advisers were 74%±1.6% accurate. Players who interacted with these advisers exhibited a learning trajectory that reflected the advisers' dynamic incentives (see Figure 7c). For example, for the representative subject shown in Figure 7c (subject SL_013), 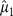 steadily reached 0.9 after the first 80 trials, with a concomitant decrease in estimates of the adviser's volatility 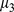. Once the adviser's intentions changed, the player updated his beliefs accordingly, as reflected by an increase in the learning rate in 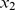 and larger updates of 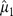. In this scenario, the player's adaptive behavior takes into account both the volatility of the adviser's intentions and the accuracy of his advice. This is reflected by high values of the estimates for κ, *ω*, and 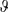.

Finally, to illustrate the capacity of our model-based approach for characterizing individual differences, we show an unusual subject in Figure 7d (subject SL_015). This player did recognize a change in the adviser's intentions halfway through the game but was much slower in updating his estimates of advice accuracy and adviser's volatility than the subject discussed above. This is because his prior beliefs were close to how the adviser actually behaved for the first half of the game and because low estimates of meta-volatility prevented a rapid response to the change in the adviser's intentions. In other words, this participant remained relatively confident about his prior estimate of the adviser's volatility and expected to see little change over the course of the social interaction.

## Discussion

The question of how we infer on others' intentions is a fundamental computational problem during social transactions. To examine this process, we extended a paradigm introduced by [14], turning it into an interactive social decision-making game in which each participant was assigned to a “player” or an “adviser” role. Critically, the game was designed to ensure that the adviser's incentives to cooperate or deceive the player varied, thus making his intentions volatile.

While our paradigm was inspired by the previous work of Behrens and colleagues [14], [42], it introduced two important advances. First, whereas Behrens et al. made subjects believe that the computer-generated advice was provided by a human being, our paradigm used real participants without any deception. This provides ecological validity and eschews potential ethical concerns, which makes the recorded trials from this paradigm more widely applicable, e.g., for future patient studies. Secondly, our paradigm allows for a wide range of interactions between agents, as both the adviser and the player are not restricted to employ specific strategies during their interaction. The player can rely on the binary lottery information (the non-social cue), the advice, or both when selecting his choices. Furthermore, on every trial, the adviser can also choose to provide either helpful or misleading advice, depending on whatever strategy he may be employing; in turn, these differences in strategy across advisers elicit differences in the adaptive behavior of the players.

To explain the ensuing variability in adaptive behavior across subjects, we modeled the players' learning using a systematic set of alternative models that factorially combined different models of learning behavior (“perceptual models”) and decision-making (“response models”). Using Bayesian model selection, we demonstrated that a hierarchical Bayesian model (the hierarchical Gaussian filter, HGF) with three levels best described the players' learning in the task. This suggests that participants updated their beliefs about advice reliability depending on an ongoing estimate of the volatility of the adviser's intentions, and that this estimate of volatility directly informed the trial-wise decisions. This three-level HGF outperformed simpler non-hierarchical models (such as Rescorla-Wagner), indicating that during social exchanges, participants employ a multilevel model of their environment and are capable of learning how others' intentions to be helpful or misleading fluctuate over time. These higher-order expectations are in turn exploited to update trial-by-trial predictions about advice reliability.

An important contribution of this paper is the translation of a recent Bayesian framework for comparing alternative cognitive models [15], [16], [22] to the domain of social interactions. The implementation of this framework in the present study, however, has one significant limitation: The present models aimed to explain only the players' learning during the game, and not the advisers'. That is, they neglected the recursive process of perspective-taking (in other words, the player's belief about the adviser's belief about the player's belief etc.), which occurs in many social situations. Recent studies (see [2], [12], [13], [43]) used recursive theory-of-mind models to explain social inference in cooperative or multi-round trust games. These models propose that the expected value of a given action (e.g., to cooperate or to compete and to choose equitable or unfair offers) is a function of the other agent's strategy. Thus, players optimize their strategies or their depth of recursive reasoning by taking into account their opponents' future actions. For example, Yoshida and colleagues (2010a) showed that players who employ higher-order strategies, which take into account the opponents' future actions, forgo immediate rewards for options that lead to higher pay-off but require multi-player cooperation.

One important future direction of our work is to extend the modeling of hierarchically coupled beliefs to take the depth of recursive perspective-taking into account. Having said this, the recursive depth of social inferences is typically limited [2], [13]. For example, Xiang et al. (2012) classified the depth of subjects' reasoning during an investor-trustee game. Approximately half of 195 investors were classified as strategic level 0 players, suggesting that they do not simulate their partner's play, while the other half employed either level 1 or level 2 depth-of-reasoning. Yoshida et al., 2010a also reported significant variability in recursive depth across individuals. In the present study, as described in the Results section, more than half (9/16) of the advisers reported to have adopted a fixed strategy (either consistently helpful or consistently random throughout the game) and are thus unlikely to have engaged in recursive perspective-taking. For the remaining advisers, it is possible that modeling their belief updating processes (in addition to those of the players) would lead to an even better prediction of the players' behavior. We will test this possibility in future work, contrasting models with and without the representation of recursive interactions.

As it did not incorporate recursive perspective-taking, our study focused on modeling the downstream consequences of the differential strategies that advisers employed. The players' belief-updating process reflected the advisers' policy and determined how much they were willing to take the advisers' suggestions into account during decision-making. We found that players who interacted with consistently helpful advisers perceived their intentions to be stable over time and thus weighted their advice more when predicting the outcome, as reflected by reduced values in the meta-volatility parameter 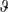 and larger 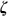 values, respectively. Furthermore, players who interacted with advisers, whose intentions were changing to maximize their own winnings, showed more pronounced 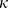 values. This result suggests that the two hierarchical learning levels were more strongly coupled in this subset of participants, and that the volatility estimate was used to update the beliefs about advice accuracy. Unlike in the case of consistently misleading advisers, in this particular social exchange, the volatility of the advisers' intentions was more traceable. Thus, players could benefit from inferring on the volatility of the advisers' intentions to predict the advice accuracy.

Beyond reflecting the adviser's policy in the parameter estimates, our model exhibited construct validity in two ways: First, its posterior parameter estimates predicted participants' scores on the IRI, a questionnaire, which they completed prior to participation in the study. Players, who described themselves as proficient perspective-takers, exhibited a more stable model of the adviser as reflected by the less pronounced tonic component of the learning rate. Second, the model's posterior parameter estimates also predicted the participants' explicit ratings of the advisers' helpfulness throughout the game. Notably, this relationship was specific for the hierarchical Bayesian model while the parameter estimates from the competing RW model did not show this predictive capacity.

As described above, model comparison indicated that the participants' behavior was best explained by a hierarchical model in which estimates of volatility (of the adviser's intentions) played a key role for belief updating. Furthermore, beyond inference and with respect to the translation of beliefs to decisions, we found that a response model in which participants' estimates of the volatility of the advisers' intentions determined their trial-wise decisions explained participants' choice behavior best. That is, the mapping from beliefs to choices was increasingly deterministic the more the player considered the adviser's intentions to be currently stable. By contrast, when the player's estimates of the adviser's volatility increased, the relation between beliefs and decisions became more stochastic and the player exhibited a more exploratory behavior. This result demonstrates the direct relevance of volatility estimates for determining trial-by-trial variability of decisions; note that this is distinct from (and complementary to) our findings on the role of volatility for learning and inference, described in the context of comparing different perceptual models above. The finding that volatility is an important factor determining trial-by-trial choice variability goes beyond previous studies, which examined the impact of volatility with respect to inference only (e.g., [14], [22], [25], [44]). Moreover, in the context of social learning, these results stress the deployment of a hierarchical model and a key role of volatility estimates for both inference and decision-making. These are important in that they complement concepts of social learning, which emphasize the role of simple heuristics (e.g., [45]) or refer to non-hierarchical reinforcement learning (e.g., [46]).

Similar to what Behrens and colleagues (2008) observed, we found that participants did not base their decision on a single source of information, but integrated the advice with information from the visual pie chart, which was also probabilistic but had a known outcome distribution. That is, the uncertainty of the information provided by the pie chart was directly given on each trial, whereas the uncertainty of the advice had to be estimated online. This can be related, to some degree, to the distinction between risk and ambiguity [47]–[49].

Our modeling results show that participants were able to trade-off between these different forms of uncertainty depending on the type of adviser they faced (see Figures 6d–e and 7): when interacting with generally helpful advisers, most players considered the advice strongly because, on average, it was more accurate than the visual pie chart. However, when they interacted with advisers who deliberately showed consistently uninformative (random) behavior, participants tended to discount their recommendation and relied more strongly on the visual pie chart. This is remarkable since it means that players did not display a uniform tendency to avoid ambiguity; instead, ambiguity aversion was restricted to interactions with an unhelpful adviser.

Additionally, we found that the different sources of information (cue and advice) did not receive equal weight during decision-making. Consistent with previous findings [50], we observed that participants relied more on the non-ambiguous information (i.e., the non-social cue) compared to the advice. Previous models [51], [52] describing how people integrate social and non-social sources emphasized the importance of ambiguity that is intrinsic to social exchange: We are uncertain about how uncertain our appraisal of the other agent's intentions is.

Previous work on uncertainty in repeated advice taking showed that, surprisingly, decision-makers do not become more confident in their choices with increasing advice accuracy [53]. Although we did not explicitly ask subjects to rate confidence or uncertainty, our modeling results did take into account how their inferred estimates of uncertainty (about the adviser's intentions) informed their trial-wise decisions.

Furthermore, analysis of a control condition in which trial-wise advice was randomly sampled (by a blindfolded adviser) from several decks of cards with either 80% or 20% accuracy suggested that participants relied more on the advice when it was intentional, as opposed to unintentional. This behavior was observed even though it was perfectly possible to extract predictive information from the card decks with the same accuracy as from a helpful adviser. Since players based their decisions more on the visual pie chart and did not take advantage of the advice, their performance was significantly lower than in the social condition.

Beyond the results per se and their implications for concepts of social learning, the modeling approach in this paper, with its emphasis on inter-individual variability in inference and decision-making, may serve useful for future studies of social learning. To facilitate this, the HGF and the BMS routines are freely available as open source MATLAB code (the HGF can be found at www.translationalneuromodeling.org/tapas; the BMS routines are part of the SPM software package: www.fil.ion.ucl.ac.uk/spm).

Finally, we believe that the approach presented here has potential for characterizing mechanisms of maladaptive behavior in individual patients. The present study in healthy volunteers provides a proof of concept how individual mechanisms can be elucidated in the context of social interactions, a domain where many psychiatric disorders, including schizophrenia, are characterized by particularly salient deficiencies [54]. For example, many patients with schizophrenia exhibit a negative attribution bias about others' intentions, which reflects the finding that negative information is perceived as more diagnostic of another person's true character than positive information [55]–[57]. One attractive option is to use models as the one described in this study for computational phenotyping of patients from heterogeneous disorders [58]–[60]. For example, patients may show a diminished ability to dynamically infer on the intentions of others for different reasons: they may have overly tight prior beliefs about others' motivations, or they may suffer from an abnormality in belief updating mechanisms, which in turn could be due to aberrant computations of prediction error, precision or both (see Eq. 6 above). In other words, models of cognition such as the one introduced here and in previous studies may prove useful to propose potential nosological dimensions with mechanistic interpretability and disambiguate alternative mechanisms in individual patients through model selection [61]. This study serves as a precursor for future neuroimaging studies, in which we hope to investigate neuronal mechanisms of social learning and tracking the volatility of another agent's intentions.

## Supporting Information

Figure S1 The performance of BMS was evaluated and the results are summarized in confusion matrices. Each cell includes the frequency with which each perceptual model wins (over simulation instances) based on data generated under each model (in rows) and inverted by itself and all other models (in columns). Thus, off-diagonal elements indicate the probability that the source of data generated by one model is “confused” with another model due to the inversion and model selection procedure.
(TIF)

Figure S2Log Bayes factors comparing the winning model (the three-level HGF augmented by the “Volatility” response model (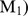) to the rest of the models across all subjects. The Bayes factors, which exceed the dotted line, (i.e., Bayes factor >100 or log evidence difference >10), represent strong evidence that the winning model outperforms the rest, according to conventional classifications (see [62]). One can see that with the exception of two subjects (SL_005 and SL_010), there is strong evidence favouring model 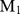 over all other models.(TIF)

Figure S3Group Bayes factors comparing the winning model (the three-level HGF augmented by the “Volatility” response model (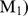) to the rest of the models. The Bayes factors, which exceed the dotted line, (i.e., Bayes factor of 100) suggest strong evidence that the winning model outperforms the rest, which exceed this threshold according to conventional classifications (see [62]).(TIF)

Figure S4Linear regression analysis of the player-specific ratings of the advisers and the model estimates: We aimed to explain participants' ratings of the advisers' intentions (dependent variable) using the estimates of advice reliability as inferred from the model (explanatory variable). The plot contains the player-specific ratings, trial-specific 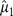 values, and the player-specific beta estimates from the first level regression analysis.(TIF)

Video S1The relationship between 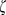 and 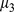 in the response model. Parameter 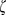 determines the weight of the advice, and 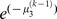 represents the inverse of the adviser phasic volatility estimate. As the inverse of 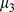 approaches ∞, the estimated volatility of the adviser's intentions decreases, and decisions are more consistent with the players' beliefs.(MOV)

## References

[1] King-Casas B, Tomlin D, Anen C, Camerer CF, Quartz SR, et al (2005) Getting to Know You: Reputation and Trust in a Two-Person Economic Exchange. Science 308: 78–83. 15802598 10.1126/science.1108062

[2] Yoshida W, Dolan RJ, Friston KJ (2008) Game Theory of Mind. PLoS Computational Biology 4: e1000254. 19112488 10.1371/journal.pcbi.1000254 PMC2596313

[3] Amodio DM, Frith CD (2006) Meeting of minds: the medial frontal cortex and social cognition. Nature Reviews Neuroscience 7: 268–277. 16552413 10.1038/nrn1884

[4] Frith CD, Frith U (2006) The Neural Basis of Mentalizing. Neuron 50: 531–534. 16701204 10.1016/j.neuron.2006.05.001

[5] Keysers C, Gazzola V (2007) Integrating simulation and theory of mind: from self to social cognition. Trends in Cognitive Sciences 11: 194–196. 17344090 10.1016/j.tics.2007.02.002

[6] Canessa N, Alemanno F, Riva F, Zani A, Proverbio AM, et al (2012) The neural bases of social intention understanding: the role of interaction goals. PLoS ONE 7: e42347. 22848759 10.1371/journal.pone.0042347 PMC3407127

[7] Ciaramidaro A, Adenzato M, Enrici I, Erk S, Pia L, et al (2007) The intentional network: how the brain reads varieties of intentions. Neuropsychologia 45: 3105–3113. 17669444 10.1016/j.neuropsychologia.2007.05.011

[8] Van Overwalle F, Baetens K (2009) Understanding others' actions and goals by mirror and mentalizing systems: a meta-analysis. Neuroimage 48: 564–584. 19524046 10.1016/j.neuroimage.2009.06.009

[9] Baker CL, Saxe RR, Tenenbaum JB (2011) Bayesian theory of mind: Modeling joint belief-desire attribution. Proceedings of the thirty-second annual conference of the cognitive science society. pp. 2469–2474.

[10] Goodman ND, Baker CL, Tenenbaum JB (2009) Cause and intent: Social reasoning in causal learning. Proceedings of the Thirty-First Annual Conference of the Cognitive Science Society. pp. 2759–2764.

[11] Ullman TD, Baker CL, Macindoe O, Evans O, Goodman ND, et al.. (2009) Help or Hinder: Bayesian Models of Social Goal Inference. NIPS. pp. 1874–1882.

[12] Devaine M, Hollard G, Daunizeau J (2014) Theory of Mind: Did Evolution Fool Us? PLoS ONE 9: e87619. 24505296 10.1371/journal.pone.0087619 PMC3914827

[13] Xiang T, Ray D, Lohrenz T, Dayan P, Montague PR (2012) Computational phenotyping of two-person interactions reveals differential neural response to depth-of-thought. PLoS Comput Biol 8: e1002841. 23300423 10.1371/journal.pcbi.1002841 PMC3531325

[14] Behrens TEJ, Hunt LT, Woolrich MW, Rushworth MFS (2008) Associative learning of social value. Nature 456: 245–U45. 19005555 10.1038/nature07538 PMC2605577

[15] Daunizeau J, den Ouden HEM, Pessiglione M, Kiebel SJ, Stephan KE, et al (2010) Observing the Observer (I): Meta-Bayesian Models of Learning and Decision-Making. PLoS One 5: e15554. 21179480 10.1371/journal.pone.0015554 PMC3001878

[16] Daunizeau J, den Ouden HEM, Pessiglione M, Kiebel SJ, Friston KJ, et al (2010) Observing the Observer (II): Deciding When to Decide. PLoS One 5: e15555. 21179484 10.1371/journal.pone.0015555 PMC3001882

[17] Huszár F, Noppeney U, Lengyel M (2010) Mind reading by machine learning: a doubly Bayesian method for inferring mental representations. Proceedings of the Thirty-Second Annual Conference of the Cognitive Science Society. pp. 2810–2815.

[18] Lee MD (2011) In praise of Ecumenical Bayes. Behavioral and Brain Sciences 34: 206–207.

[19] Buchan NR, Croson RTA, Solnick S (2008) Trust and gender: An examination of behavior and beliefs in the Investment Game. Journal of Economic Behavior & Organization 68: 466–476.

[20] Cloninger CR, Svrakic DM, Przybeck TR (1993) A psychobiological model of temperament and character. Archives of general psychiatry 50: 975–990. 8250684 10.1001/archpsyc.1993.01820240059008

[21] Davis MH (1983) Measuring individual differences in empathy: Evidence for a multidimensional approach. Journal of Personality and Social Psychology 44: 113–126.

[22] Mathys C, Daunizeau J, Friston KJ, Stephan KE (2011) A Bayesian foundation for individual learning under uncertainty. Front Hum Neurosci 5: 1–20. 21629826 10.3389/fnhum.2011.00039 PMC3096853

[23] Rescorla RA, Wagner AR (1972) A theory of Pavlovian conditioning: Variations in the effectiveness of reinforcement. New York: Appleton-Century-Crofts.

[24] Vossel S, Mathys C, Daunizeau J, Bauer M, Driver J, et al (2014) Spatial Attention, Precision, and Bayesian Inference: A Study of Saccadic Response Speed. Cereb Cortex 24: 1436–1450. 23322402 10.1093/cercor/bhs418 PMC4014178

[25] Iglesias S, Mathys C, Brodersen KH, Kasper L, Piccirelli M, et al (2013) Hierarchical Prediction Errors in Midbrain and Basal Forebrain during Sensory Learning. Neuron 80: 519–530. 24139048 10.1016/j.neuron.2013.09.009

[26] Doya K, Ishii S, Pouget A, Rao RPN (2011) Bayesian brain: probabilistic approaches to neural coding. Cambridge, Mass.: MIT Press.

[27] Friston K (2010) The free-energy principle: a unified brain theory? Nat Rev Neurosci 11: 127–138. 20068583 10.1038/nrn2787

[28] Griffiths TL, Kemp C, Tenenbaum JB (2008) Bayesian models of cognition. Cambridge handbook of computational cognitive modeling: 59–100.

[29] Körding K (2007) Decision Theory: What “Should” the Nervous System Do? Science 318: 606–610. 17962554 10.1126/science.1142998

[30] Tenenbaum JB, Kemp C, Griffiths TL, Goodman ND (2011) How to Grow a Mind: Statistics, Structure, and Abstraction. Science 331: 1279–1285. 21393536 10.1126/science.1192788

[31] Montague PR, Hyman SE, Cohen JD (2004) Computational roles for dopamine in behavioural control. Nature 431: 760–767. 15483596 10.1038/nature03015

[32] Preuschoff K, Bossaerts P (2007) Adding Prediction Risk to the Theory of Reward Learning. Annals of the New York Academy of Sciences 1104: 135–146. 17344526 10.1196/annals.1390.005

[33] Broyden CG (1970) The Convergence of a Class of Double-rank Minimization Algorithms 1. General Considerations. IMA J Appl Math 6: 76–90.

[34] Fletcher R (1970) A new approach to variable metric algorithms. The Computer Journal 13: 317–322.

[35] Goldfarb D (1970) A family of variable metric methods derived by variational means. Mathematics of computation 24: 23–26.

[36] Shanno DF (1970) Conditioning of quasi-Newton methods for function minimization. Mathematics of computation 24: 647–656.

[37] Osborne MA, Garnett R, Roberts SJ (2009) Gaussian processes for global optimization. 3rd international conference on learning and intelligent optimization (LION3). pp. 1–15.

[38] Hastings WK (1970) Monte Carlo sampling methods using Markov chains and their applications. Biometrika 57: 97–109.

[39] Penny WD, Stephan KE, Daunizeau J, Rosa MJ, Friston KJ, et al (2010) Comparing families of dynamic causal models. PLoS computational biology 6: e1000709. 20300649 10.1371/journal.pcbi.1000709 PMC2837394

[40] MacKay DJ (2003) Information theory, inference and learning algorithms. Cambridge university press.

[41] Stephan KE, Penny WD, Daunizeau J, Moran RJ, Friston KJ (2009) Bayesian model selection for group studies. NeuroImage 46: 1004–1017. 19306932 10.1016/j.neuroimage.2009.03.025 PMC2703732

[42] Behrens TEJ, Hunt LT, Rushworth MFS (2009) The Computation of Social Behavior. Science 324: 1160–1164. 19478175 10.1126/science.1169694

[43] Yoshida W, Seymour B, Friston KJ, Dolan RJ (2010a) Neural Mechanisms of Belief Inference during Cooperative Games. J Neurosci 30: 10744–10751. 20702705 10.1523/JNEUROSCI.5895-09.2010 PMC2967416

[44] Behrens TEJ, Woolrich MW, Walton ME, Rushworth MFS (2007) Learning the value of information in an uncertain world. Nat Neurosci 10: 1214–1221. 17676057 10.1038/nn1954

[45] Hertwig R, Hoffrage U, Group AR (2012) Simple Heuristics in a Social World. Oxford University Press. 662 p.

[46] Biele G, Rieskamp J, Krugel LK, Heekeren HR (2011) The Neural Basis of Following Advice. Plos Biology 9: e1001089. 21713027 10.1371/journal.pbio.1001089 PMC3119653

[47] Charness G, Karni E, Levin D (2013) Ambiguity attitudes and social interactions: An experimental investigation. J Risk Uncertain 46: 1–25.

[48] Chow CC, Sarin RK (2002) Known, Unknown, and Unknowable Uncertainties. Theory and Decision 52: 127–138.

[49] Ellsberg D (1961) Risk, Ambiguity, and the Savage Axioms. The Quarterly Journal of Economics 75: 643–669.

[50] Soll JB, Larrick RP (2009) Strategies for revising judgment: How (and how well) people use others' opinions. Journal of Experimental Psychology: Learning, Memory, and Cognition 35: 780–805. 10.1037/a0015145 19379049

[51] Collins EC, Percy EJ, Smith ER, Kruschke JK (2011) Integrating advice and experience: Learning and decision making with social and nonsocial cues. Journal of Personality and Social Psychology 100: 967–982. 21443371 10.1037/a0022982

[52] Van Overwalle F, Heylighen F (2006) Talking nets: A multiagent connectionist approach to communication and trust between individuals. Psychological Review 113: 606–627. 16802883 10.1037/0033-295X.113.3.606

[53] Lee MD, Dry MJ (2006) Decision Making and Confidence Given Uncertain Advice. Cognitive Science 30: 1081–1095. 21702847 10.1207/s15516709cog0000_71

[54] Kishida KT, Montague PR (2012) Imaging Models of Valuation During Social Interaction in Humans. Biological Psychiatry 72: 93–100. 22507699 10.1016/j.biopsych.2012.02.037 PMC3544196

[55] Bentall RP, Corcoran R, Howard R, Blackwood N, Kinderman P (2001) Persecutory delusions: A review and theoretical integration. Clinical Psychology Review 21: 1143–1192. 11702511 10.1016/s0272-7358(01)00106-4

[56] Fett AK, Shergill SS, Joyce DW, Riedl A, Strobel M, et al (2012) To trust or not to trust: the dynamics of social interaction in psychosis. Brain 135: 976–984. 22366802 10.1093/brain/awr359

[57] Langdon R, Corner T, McLaren J, Ward PB, Coltheart M (2006) Externalizing and personalizing biases in persecutory delusions: The relationship with poor insight and theory-of-mind. Behaviour Research and Therapy 44: 699–713. 16038873 10.1016/j.brat.2005.03.012

[58] King-Casas B, Sharp C, Lomax-Bream L, Lohrenz T, Fonagy P, et al (2008) The Rupture and Repair of Cooperation in Borderline Personality Disorder. Science 321: 806–810. 18687957 10.1126/science.1156902 PMC4105006

[59] Montague PR, Dolan RJ, Friston KJ, Dayan P (2012) Computational psychiatry. Trends in Cognitive Sciences 16: 72–80. 22177032 10.1016/j.tics.2011.11.018 PMC3556822

[60] Yoshida W, Dziobek I, Kliemann D, Heekeren HR, Friston KJ, et al (2010b) Cooperation and heterogeneity of the autistic mind. The Journal of neuroscience 30: 8815–8818. 20592203 10.1523/JNEUROSCI.0400-10.2010 PMC2907513

[61] Stephan KE, Mathys CD (2014) Computational Approaches to Psychiatry. Current Opinion in Neurobiology 25: 85–92. 24709605 10.1016/j.conb.2013.12.007

[62] Kass RE, Raftery AE (1995) Bayes Factors. Journal of the American Statistical Association 90: 773–795.

