# Correction: Inferring on the Intentions of Others by Hierarchical Bayesian Learning

**DOI:** 10.1371/journal.pcbi.1003952

**Published:** 2014-10-16

**Authors:** The PLOS Computational Biology Staff

There is an affiliation missing for the second author Christoph Mathys. The full list of affiliations for this author should read:

Translational Neuromodeling Unit (TNU), Institute for Biomedical Engineering, University of Zurich and ETH Zurich, Zurich, Switzerland,

Laboratory for Social and Neural Systems Research, Department of Economics, University of Zurich, Zurich, Switzerland,

Wellcome Trust Centre for Neuroimaging, University College London, London, United Kingdom

Max Planck UCL Centre for Computational Psychiatry and Ageing Research, London, United Kingdom
