# Supplementary figures and images for: Inferring on the Intentions of Others by Hierarchical Bayesian Learning
Source: PLoS Comput Biol. 2014 Sep 4;10(9):e1003810. doi: 10.1371/journal.pcbi.1003810 (PMC4154656; doi:10.1371/journal.pcbi.1003810)

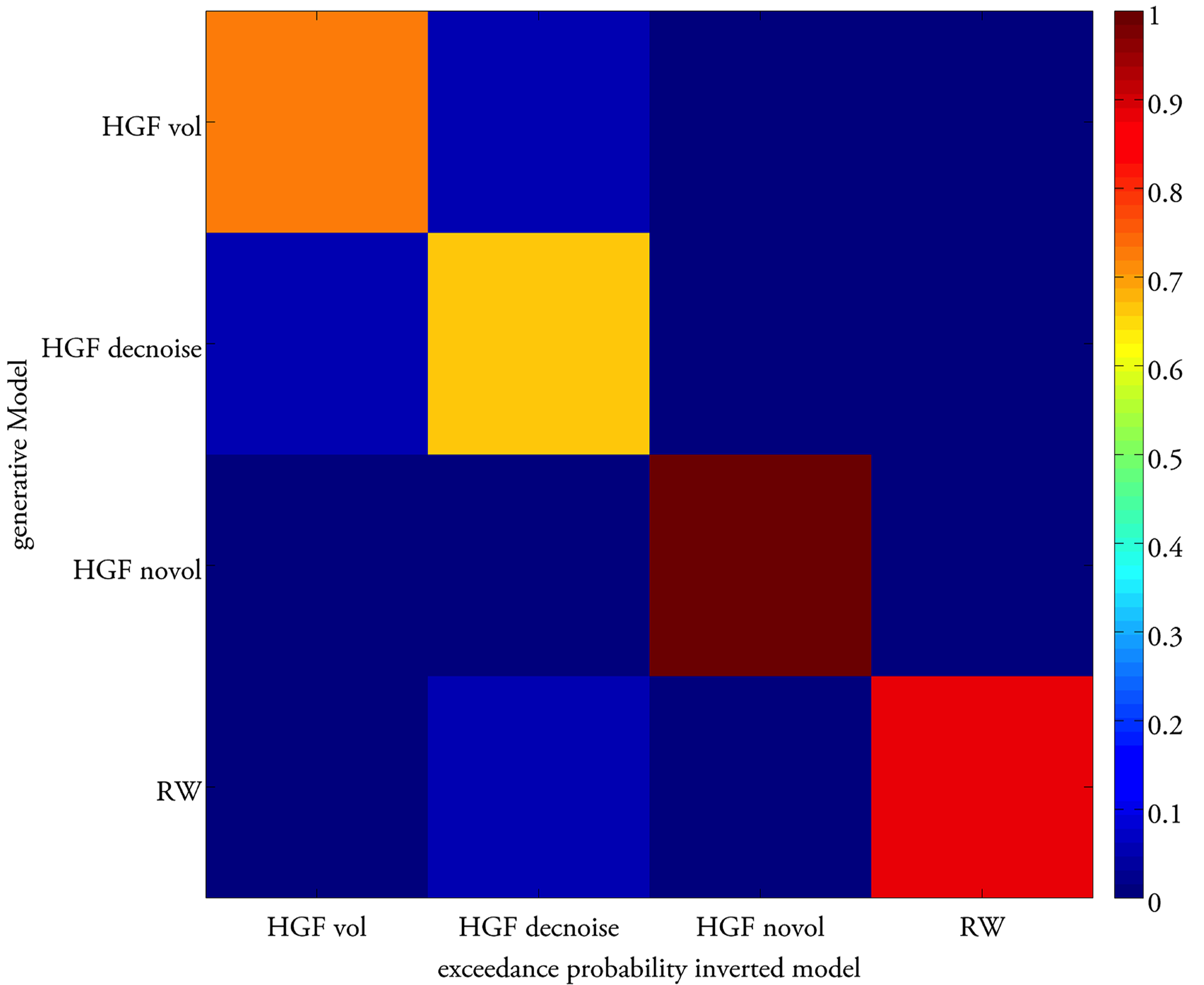

Supplement: Figure S1 — The performance of BMS was evaluated and the results are summarized in confusion matrices. Each cell includes the frequency with which each perceptual model wins (over simulation instances) based on data generated under each model (in rows) and inverted by itself and all other models (in columns). Thus, off-diagonal elements indicate the probability that the source of data generated by one model is “confused” with another model due to the inversion and model selection procedure. (TIF) [file pcbi.1003810.s001.tif]

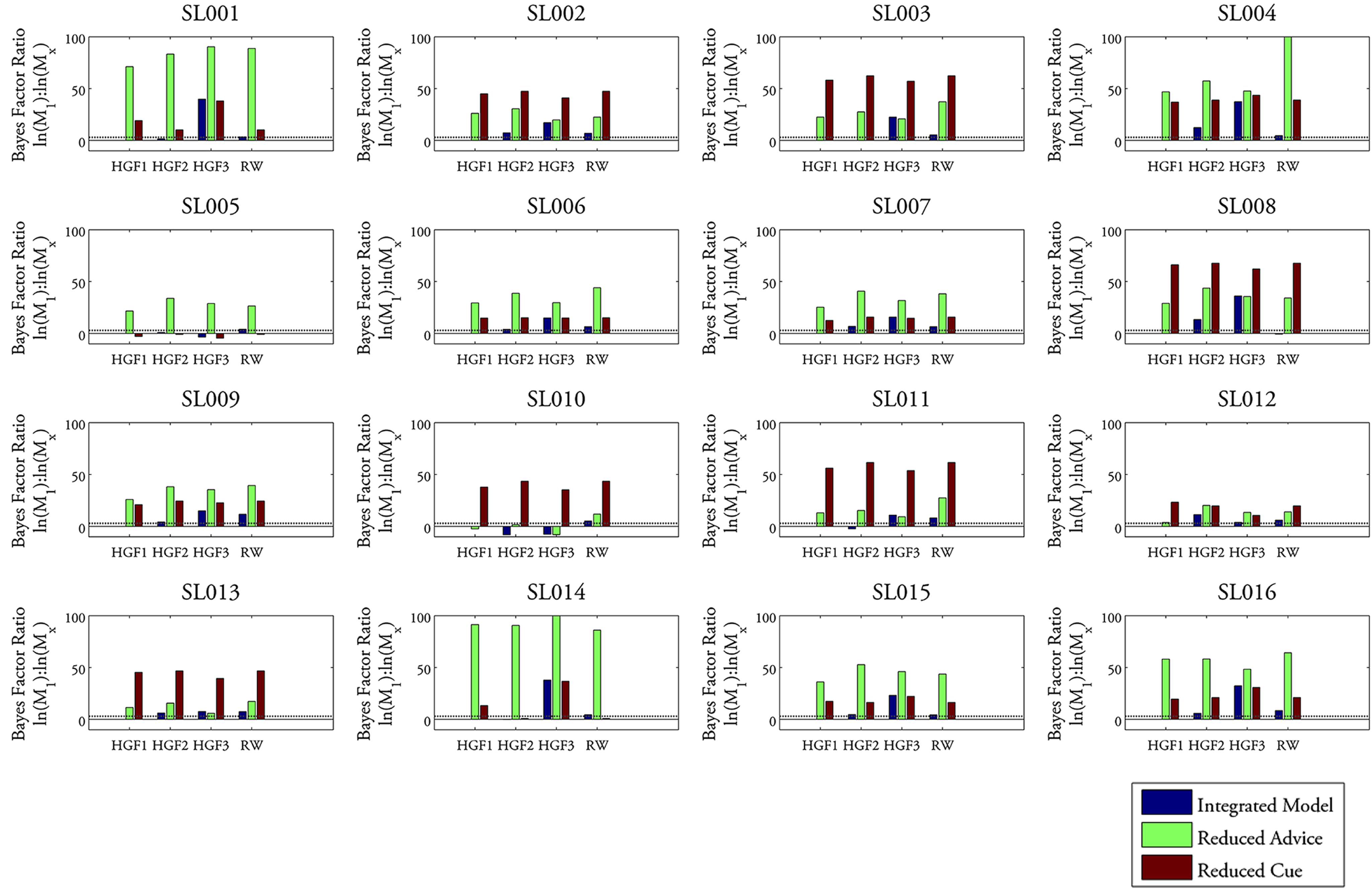

Supplement: Figure S2 — Log Bayes factors comparing the winning model (the three-level HGF augmented by the “Volatility” response model () to the rest of the models across all subjects. The Bayes factors, which exceed the dotted line, (i.e., Bayes factor >100 or log evidence difference >10), represent strong evidence that the winning model outperforms the rest, according to conventional classifications (see [62]). One can see that with the exception of two subjects (SL_005 and SL_010), there is strong evidence favouring model over all other models. (TIF) [file pcbi.1003810.s002.tif]

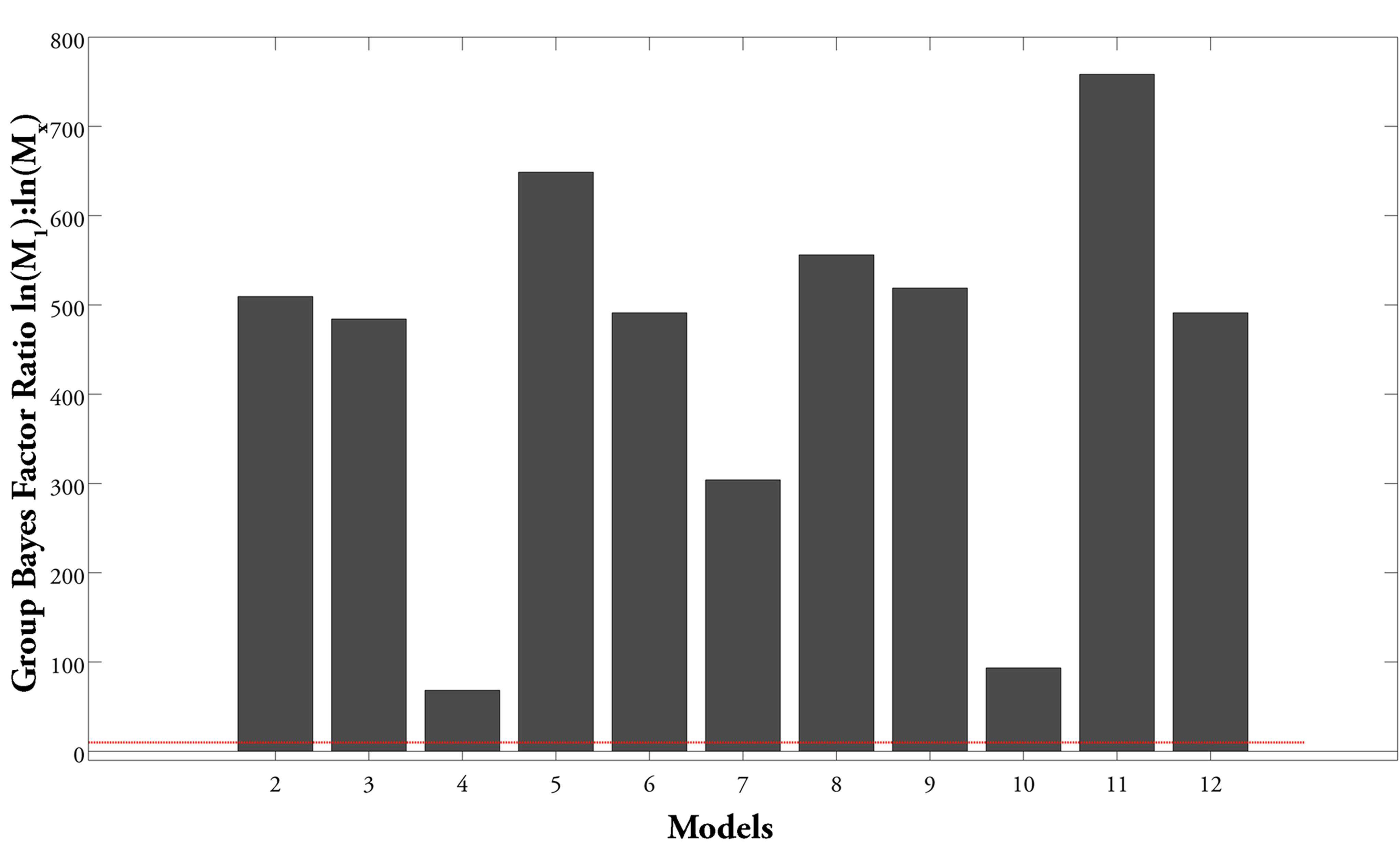

Supplement: Figure S3 — Group Bayes factors comparing the winning model (the three-level HGF augmented by the “Volatility” response model () to the rest of the models. The Bayes factors, which exceed the dotted line, (i.e., Bayes factor of 100) suggest strong evidence that the winning model outperforms the rest, which exceed this threshold according to conventional classifications (see [62]). (TIF) [file pcbi.1003810.s003.tif]

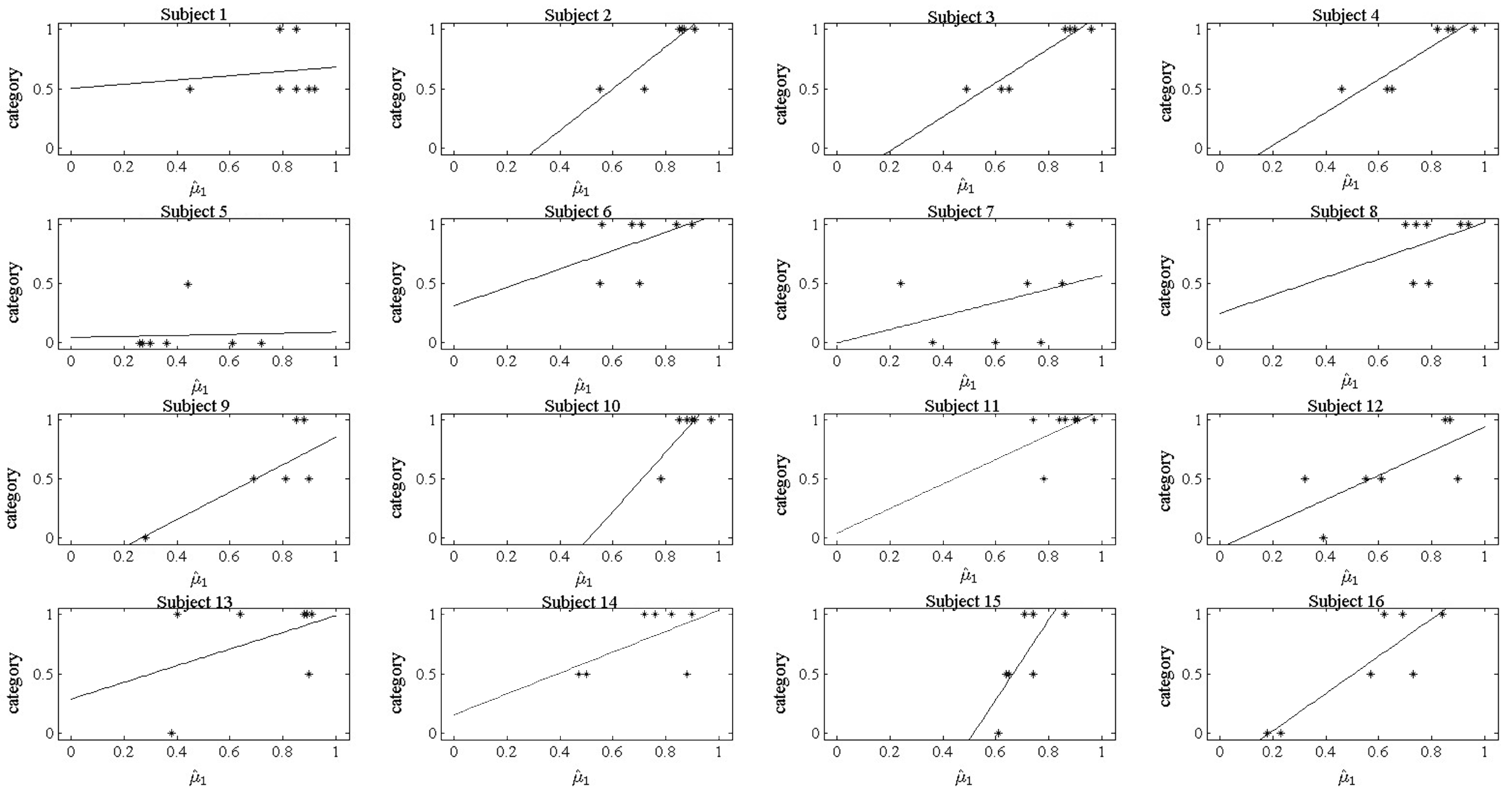

Supplement: Figure S4 — Linear regression analysis of the player-specific ratings of the advisers and the model estimates: We aimed to explain participants' ratings of the advisers' intentions (dependent variable) using the estimates of advice reliability as inferred from the model (explanatory variable). The plot contains the player-specific ratings, trial-specific values, and the player-specific beta estimates from the first level regression analysis. (TIF) [file pcbi.1003810.s004.tif]
